# Higher thresholds for the utilization of steatotic allografts in liver transplantation: Analysis from a U.S. national database

**DOI:** 10.1371/journal.pone.0230995

**Published:** 2020-04-02

**Authors:** Justin A. Steggerda, Matthew B. Bloom, Mazen Noureddin, Todd V. Brennan, Tsuyoshi Todo, Nicholas N. Nissen, Andrew S. Klein, Irene K. Kim

**Affiliations:** 1 Department of Surgery, Cedars-Sinai Medical Center, Los Angeles, California, United States of America; 2 Division of Trauma and Critical Care Surgery, Cedars-Sinai Medical Center, Los Angeles, California, United States of America; 3 Department of Hepatology, Cedars-Sinai Medical Center, Los Angeles, California, United States of America; 4 Division of Transplant Surgery, Cedars-Sinai Medical Center, Los Angeles, California, United States of America; Harvard Medical School, UNITED STATES

## Abstract

**Background:**

Historically, liver allografts with >30% macrosteatosis (MaS) on donor biopsy have been associated with early allograft dysfunction and worse graft survival; however, successful outcomes have been reported in small cohorts. This study proposes an elevated MaS threshold for organ utilization without detriment to graft survival.

**Methods:**

The UNOS Standard Transplant Analysis and Research database was evaluated for transplants between 2006–2015. Graft survival up to 1-year was evaluated by Kaplan-Meier (KM) survival analyses, and by univariate and multivariable logistic regression analyses, including donor and recipient characteristics. Odds ratios (OR) with 95% confidence intervals (CI) for risk of graft loss are reported.

**Results:**

Thirty-day risk of graft loss was increased with MaS as low as 10–19% (OR [95% CI] 1.301 [1.055–1.605], p<0.0001) and peaked with MaS 50–59% (2.921 [1.672–5.103]). At 1-year, risk of graft loss remained elevated with MaS 40–49% (1.465 [1.002–2.142]) and MaS 50–59% (1.978 [1.281–3.056], p = 0.0224). Multivariable models were created for Lower and Higher MELD recipients and MaS cutoffs were established. In Lower MELD recipients, organs with ≥50% MaS had increased risk of graft loss at 30 days (2.451 [1.541–3.897], p = 0.0008) and 1-year post-transplant (1.720 [1.224–2.418], p = 0.0125). Higher MELD recipients had increased risk of graft loss at 30 days with allografts showing MaS ≥40% (4.204 [1.440–5.076], p = 0.0016). At 1-year the risk remained increased, but MaS was not significant predictor of graft loss.048 [1.131–3.710], p = 0.0616). In both MELD cohorts, organs with MaS levels below threshold had similar survival to those transplanted without a donor biopsy.

**Conclusions:**

In conjunction with recipient selection, organs with MaS up to 50% may be safely used without detriment to outcomes.

## Introduction

Currently, over 13,000 candidates are listed for and awaiting liver transplantation nationwide (UNOS Data as of April 3, 2019). Despite a steady increase in the number of liver transplants occurring in the United States over the past five years[1], there remains a significant unmet need for donor organs. Strategies to increase donor utilization have promoted the use marginal or extended criteria donors (ECD), including those with hepatic steatosis.[2]

Hepatic steatosis is common, being reported in 30–51% of donor livers,[3,4] a number that may increase with rising prevalence of obesity and non-alcoholic fatty liver disease in the United States.[5,6] Liver steatosis is characterized as either microvesicular (MiS) or macrovesicular (MaS); however, only MaS has been shown to significantly influence allograft and patient survival following liver transplantation (LT).[7–10]The volume of hepatic MaS is based on histologic evaluation and classically described as mild (<30%), moderate (30–60%), or severe (>60%).

The use of grafts with mild steatosis is generally accepted, as Kwon *et al*. demonstrated successful outcomes using donor allografts with <30% MaS.[11] Conversely, increased MaS in donor livers has been associated with early allograft dysfunction (EAD), primary graft non-function (PNF), and biliary complications.[8,12] Spitzer *et al*. showed an increased risk ratio (RR) of 1.71 for graft loss at 1-year with allografts having >30% MaS.[13] In contrast, McCormack *et al*. found increased rates of primary graft dysfunction, but no difference in 60-day or 3-year patient mortality using allografts with severe steatosis.[14] Multiple clinical series have reported similarly acceptable outcomes with the use of allografts with higher volumes of MaS.[15–23] These data are derived from small cohorts at single institutions and no large or nationwide studies exist, leaving significant controversy over the utilization of allografts with elevated MaS.

As waitlist registrations continue to rise, ongoing efforts to expand the donor pool are imperative. Using the Organ Procurement and Transplant Network Standard Transplant Analysis and Research (OPTN STAR) database, we hypothesized that liver allografts with increased levels of MaS above 30% may be used safely in LT. Furthermore, we attempted to identify a threshold MaS level for safe transplantation and characterize the donor and recipient factors which contribute to short and long-term graft survival amongst steatotic allografts in the modern transplant era.

## Methods

### Patient population

The OPTN STAR database contains prospectively collected data on donors and recipients for transplantation of all organs. A retrospective review of liver transplants and potential liver donors between January 1, 2006 and June 30, 2015 was performed. Data were obtained from the OPTN directly and are available to all interested parties through the OPTN (www.optn.transplant.hrsa.gov). Institutional approval was sought from the Cedars-Sinai Medical Center review board and given the de-identified nature of the data, it was deemed that additional IRB approval was not necessary.

Data were evaluated and exclusion criteria were applied. Exclusion criteria included pediatric patients (<18 years-old), multi-organ or split-liver recipients, donation after circulatory death (DCD) transplants, recipients with prior liver transplant, recipients listed as Status 1A, as well as any recipient without at least 1-year of follow-up. After applying these criteria, 41,347 transplants were available for analysis. Pre-donation liver biopsy was identified in 19,137 transplants, of which 16,306 had information on donor liver steatosis and comprised the study group.

All transplants with donor liver biopsy were categorized by volume of MaS identified on biopsy. Seven MaS groups were identified: 0–9% (n = 9,999 transplants), 10–19% (n = 2,673), 20–29% (n = 1,122), 30–39% (n = 762), 40–49% (n = 223), 50–59% (n = 150), and ≥60% (n = 142). Organs without a donor liver biopsy were classified as “No Biopsy.” Donor liver biopsy is typically used in the evaluation of questionable donors, while donors without a biopsy are considered acceptable to transplant for selected recipients. Therefore, these donors comprise an “ideal” control group by which to compare outcomes.

Donor and recipient characteristics were collected and evaluated, including age, gender, ethnicity, blood type, body mass index (BMI), viral status for Epstein-Barr (EBV), cytomegalovirus (CMV), hepatitis B virus (HBV) and hepatitis C virus (HCV), as well as cold ischemic time (CIT). Donor-specific variables included biopsy results with percent MaS, calculated Donor Risk Index (DRI), history of hypertension, diabetes, prior myocardial infarctions, history of cigarette or drug use, and Center for Disease Control (CDC) classification as high-risk donor. Recipient-specific variables included lab-based Model for End-stage Liver Disease (MELD) score, etiology of liver disease, history of diabetes, prior abdominal surgery, portal vein (PV) thrombosis, prior transjugular intrahepatic portosystemic shunt (TIPS), and need for dialysis or mechanical ventilation at time of transplant.

### Outcomes

The primary outcome was graft survival following transplantation. Graft survival was defined as the period between transplantation and graft loss necessitating re-transplantation or recipient death, whichever occurred first. Graft survival was assessed for the duration of individual patient follow-up, up to 1-year after transplantation. Patients who were lost to follow-up with a functioning graft were censored.

Subgroup analyses were performed with subgroup determination based on recipient MELD score. Two subgroups were created—Lower MELD (score <33) and Higher MELD (score 33 to 40). For easier application in the current clinical scene, grouping determination was based on recently implemented allocation policies, which facilitate local-regional sharing of available allografts amongst recipients with MELD scores of 33 and higher.

### Statistical analysis

Statistical analysis was performed in JMP Pro 13.1 (SAS Institute Inc., Cary, NC). Single variable analyses were performed using Students t-tests and ANOVA where appropriate; multiple variable categorical analyses were performed using Pearson’s chi-squared tests. Kaplan-Meier (KM) survival analyses were performed and were censored for patients with functioning graft at last known follow-up with survival times truncated to 365 days. Logistic regression models were utilized to evaluate graft survival at 30 days, 90 days, and 1-year post-transplant. Univariable analyses identified factors for inclusion in multivariable analyses using a p-value <0.10 as a threshold for inclusion. A manual, stepwise regression analysis was performed using a threshold p-value of <0.05 for significance with the exception of inclusion for factors previously shown to influence graft survival (ie. donor age, recipient age, etc).[24] Odds ratios (OR) and 95% confidence intervals (CI) for graft loss are reported. Initial models were created including only transplants in which pre-donation liver biopsy had been performed. The variables of region of transplant (eg. UNOS region 1 to 11), donor and recipient age, and recipient etiology of end-stage liver disease were forced into models in which they were not otherwise included based on significance, given their import in clinical decision making. P-values are reported per Wilcoxon-Rank sum for graft survival at 1-year. Findings were considered significant with p-value <0.05. Bonferroni corrections were applied for multiple comparisons.

## Results

### Population characteristics

After applying exclusion criteria, 41,347 transplants remained for analysis. Donor liver biopsy was performed in 16,306 (39.4%), comprising the biopsy cohort. Select donor and recipient characteristics are presented in Table 1 (complete characteristics in S1 Table). Briefly, allografts with a biopsy were from donors who were older, had higher BMI, and overall a higher calculated DRI. Recipients of organs with a biopsy were slightly older and a smaller proportion of biopsied allografts went to Higher MELD recipients, recipients on a ventilator, or those with dialysis within one week of transplant.

**Table 1:** Selected donor and recipient characteristics for transplants with and without donor liver biopsy.

	All Transplants (n = 41,347)	Donor Biopsy (n = 16,306)	No Biopsy (n = 25,041)	p-Value
Donor Characteristics				
Age, years (mean±SD, median (IQR))	43.0 ± 16.8	50.0 ± 14.9	38.4 ± 16.3	<0.001
	44 (28–56)	51 (40–61)	38 (24–51)	
Gender (female)	16,891 (40.9%)	7,555 (46.3%)	9,336 (37.3%)	<0.001
Ethnicity				<0.001
White	27,187 (65.8%)	11,080 (68.0%)	16,107 (64.3%)	
Black	7,555 (18.3%)	3,017 (18.5%)	4,538 (18.1%)	
Hispanic	5,047 (12.2%)	1,591 (9.8%)	3,456 (13.8%)	
Asian	1,024 (2.5%)	397 (2.4%)	627 (2.5%)	
Other	534 (1.3%)	221 (1.4%)	313 (1.3%)	
BMI, kg/m^2^	27.7 ± 6.3	29.5 ± 7.3	26.4 ± 5.3	<0.001
Cause of Death				<0.001
Anoxia	9,566 (23.1%)	4,225 (25.9%)	5,341 (21.3%)	
Trauma	13,622 (33.0%)	3,537 (21.7%)	10,085 (40.3%)	
CVA	17,142 (41.5%)	8,164 (50.1%)	8,978 (35.9%)	
Other	1,017 (2.5%)	380 (2.3%)	637 (2.5%)	
Diabetes	5,153 (12.5%)	3,159 (19.5%)	1,994 (8.0%)	<0.001
Hypertension	15,663 (38.1%)	8,448 (52.2%)	7,215 (29.0%)	<0.001
Cigarette Smoker	11,129 (26.9%)	5,587 (34.3%)	5,542 (22.1%)	<0.001
Any Drug Use	15,524 (37.6%)	5,705 (35.0%)	9,819 (37.6%)	<0.001
HCV-Positive	1,747 (4.2%)	1,366 (8.4%)	381 (1.5%)	<0.001
HBV-Positive	2,396 (5.8%)	1,485 (9.1%)	911 (3.6%)	<0.001
Donor Risk Index	1.75 ± 0.39	1.89 ± 0.41	1.66 ± 0.34	<0.001
CIT Groups				<0.001
<8 hours	29,325 (72.3%)	10,839 (67.6%)	18,486 (75.4%)	
8 to 12 hours	9,676 (23.9%)	4,484 (28.0%)	5,192 (21.2%)	
≥12 hours	1,560 (3.9%)	712 (4.4%)	848 (3.5%)	
Recipient Characteristics				
Age	55.2 ± 9.4	55.7 ± 9.0	54.9 ± 9.7	<0.001
	56 (51–61)	57 (51–62)	56 (50–61)	
Gender (female)	12,722 (30.8%)	4,719 (28.9%)	8,003 (32.0%)	<0.001
Ethnicity				<0.001
White	29,686 (71.8%)	12,091 (74.2%)	17,595 (70.3%)	
Black	3,763 (9.1%)	1,466 (9.0%)	2,297 (9.2%)	
Hispanic	5,508 (13.3%)	1,849 (11.3%)	3,659 (14.6%)	
Asian	1,883 (4.6%)	672 (4.1%)	1,211 (4.8%)	
Other	507 (1.2%)	228 (1.4%)	279 (1.1%)	
MELD Score	22 (15–30)	21 (15–29)	23 (16–31)	<0.001
MELD Groups				<0.001
Lower MELD (Score <33)	33,586 (81.3%)	13,840 (85.0%)	19,746 (78.9%)	
Higher MELD (Score 33–40)	7,710 (18.7%)	2,441 (15.0%)	5,269 (21.1%)	
Prior TIPS	3,773 (9.3%)	1,519 (9.5%)	2,254 (9.1%)	0.29
PV Thrombosis	4,160 (10.2%)	1,625 (10.1%)	2,535 (10.3%)	0.57
Encephalopathy	25,757 (62.3%)	9,873 (60.6%)	15,884 (63.4%)	<0.001
Ascites	31,251 (75.6%)	12,096 (74.2%)	19,155 (76.5%)	<0.001
Dialysis within 1 week of Transplant	3,372 (8.2%)	1,018 (6.3%)	2,354 (9.4%)	<0.001

Graft survival was compared between recipients of liver allografts with and without donor liver biopsy. Overall, graft survival was lower for biopsied organs than those without a biopsy at 30 days (95.6% vs 96.4%, p<0.001), 90 days (93.2% vs 94.1%, p<0.001), and 1-year (86.5% vs 87.9%, p<0.001) after transplant. Interestingly, amongst allografts that survived 30 days, there was no difference in 90-day survival (97.6% with biopsy vs 97.6% without biopsy, p = 0.82) and a small but statistically significant difference in 1-year survival (90.8% with biopsy vs 91.4% without biopsy, p = 0.044).

### Graft loss following donor biopsy

**Fig 1:**
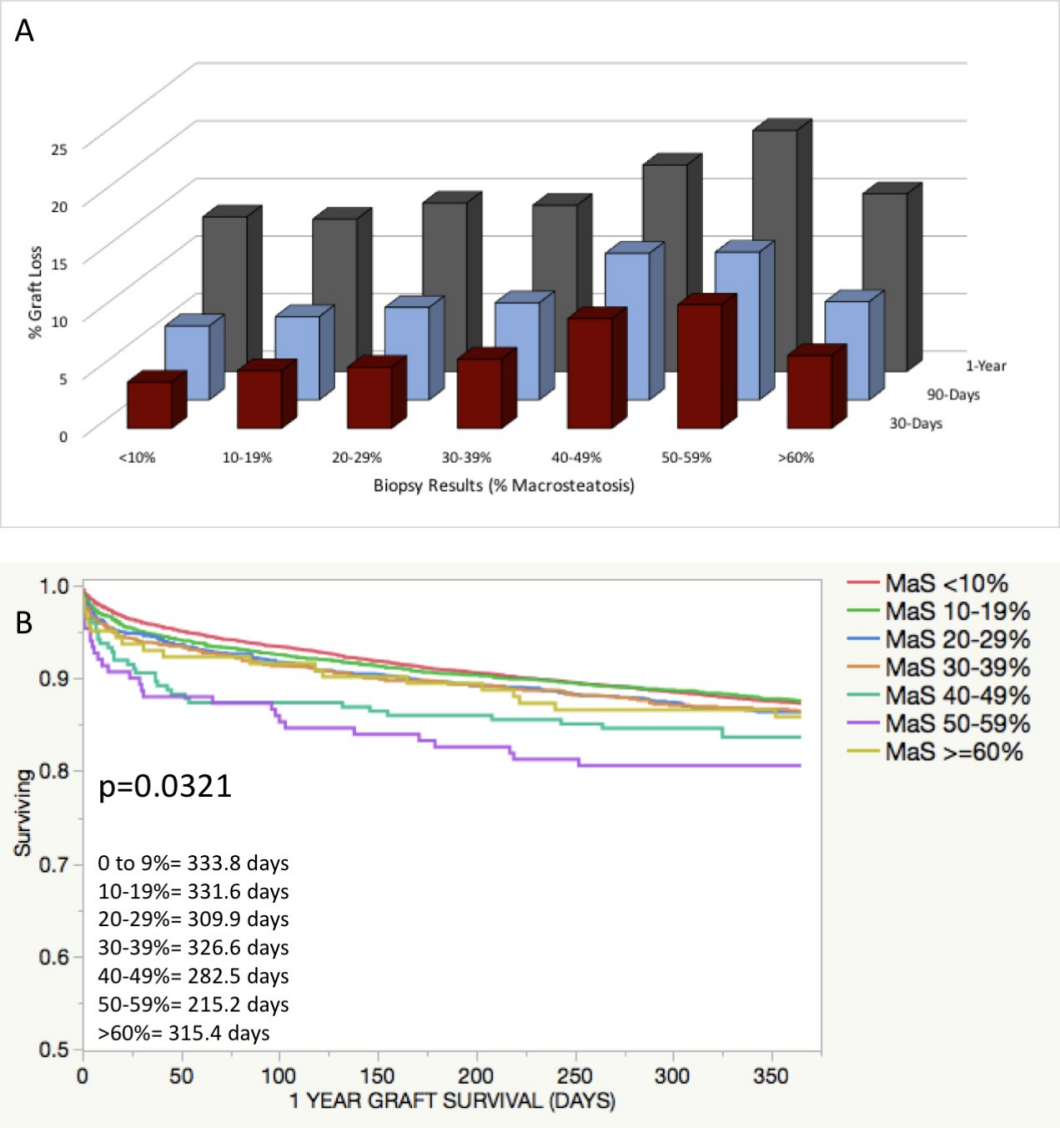
Allograft survival following donor liver biopsy by % macrosteatosis. Organs with donor liver biopsy were grouped by % MaS observed on biopsy. A) Incidence of graft loss is reported by MaS group at 30 days, 90 days and 1-year after transplant. P-value <0.0001 assessed for each follow-up time point comparing across MaS groups. B) KM survival analysis performed for 1-year graft survival amongst all organs with donor liver biopsy, comparing MaS groups. Mean graft survival reported; all graft survival to 365 days.

Incidence of graft loss increased with increasing volumes of steatosis (Fig 1A). At 30 days post-transplant, incidence of graft loss was lowest amongst allografts with 0–9% MaS (4.0%) and was highest with MaS 40–49% (9.5%) and MaS 50–59% (10.7%, p<0.001). Similar trends were seen at 90 days and 1-year follow-up. At 1-year, incidence of graft loss was highest for MaS 40–49%, 50–59% and ≥60% groups (17.9%, 20.9%, and 15.4%; p<0.001). KM survival analysis of organs with biopsy showed significant differences in graft survival across biopsy groups (Fig 1B). Interestingly, grafts with ≥60% had better graft survival than those with 40–49% and 50–59%, which likely represents a selection bias for these very high MaS organs.

Donor and recipient characteristics within each MaS group are presented in Table 2 (complete characteristics in S2 Table). Higher MaS donors were younger (p<0.001) and had lower rates of diabetes (p = 0.01) and hypertension (p<0.001); however, DRI was similar across groups (p = 0.11). Notably, there were no significant differences in recipient age, ethnicity, BMI, etiology of ESLD, or MELD score across MaS groups.

**Table 2:** Donor and recipient characteristics by donor biopsy MaS group.

	0–9% MaS (n = 9,999)	10–19% MaS (n = 2,673)	20–29% MaS (n = 1,122)	30–39% MaS (n = 762)	40–49% MaS (n = 223)	50–59% MaS (n = 150)	≥60% MaS (n = 142)	p-Value
**Donor Characteristics**								
Age, years (mean±SD)	50.0 ± 15.4	51.5 ± 13.9	50.2 ± 13.1	50.1 ± 13.1	46.2 ± 13.6	43.5 ± 12.2	46.0 ± 14.2	**<0.001**
Gender (female)	4,661 (46.6%)	1,264 (47.3%)	490 (43.7%)	339 (44.5%)	88 (39.5%)	73 (48.7%)	71 (50.0%)	0.09
Ethnicity								**<0.001**
White	6,751 (67.5%)	1,843 (69.0%)	781 (69.6%)	535 (70.2%)	152 (68.2%)	102 (68.0%)	104 (73.2%)	
Black	2,055 (20.6%)	404 (15.1%)	149 (13.3%)	104 (13.7%)	17 (7.6%)	14 (9.3%)	23 (16.2%)	
Hispanic	826 (8.3%)	311 (11.6%)	136 (12.1%)	93 (12.2%)	48 (21.5%)	30 (20.0%)	12 (8.5%)	
Asian	237 (2.4%)	77 (2.9%)	32 (2.9%)	20 (2.6%)	2 (0.9%)	3 (2.0%)	2 (1.4%)	
Other	130 (1.3%)	38 (1.4%)	24 (2.1%)	10 (1.3%)	4 (1.8%)	1 (0.7%)	1 (0.7%)	
BMI, kg/m^2^	28.7 ± 7.2	31.0 ± 7.3	31.9 ± 7.2	32.1 ± 7.4	31.7 ± 7.1	30.7 ± 7.4	30.8 ± 7.2	**<0.001**
Diabetes	1,911 (19.2%)	569 (21.4%)	219 (19.7%)	160 (21.1%)	41 (18.6%)	17 (11.5%)	20 (14.3%)	**0.01**
Hypertension	5,115 (51.5%)	1,434 (54.0%)	611 (55.2%)	441 (58.3%)	98 (44.3%)	58 (39.5%)	63 (45.0%)	**<0.001**
HCV-Positive	959 (9.6%)	172 (6.4%)	66 (5.9%)	25 (3.3%)	6 (2.7%)	13 (8.7%)	4 (2.8%)	**<0.001**
Donor Risk Index (median, IQR)	1.85 (1.57–2.16)	1.86 (1.61–2.16)	1.85 (1.59–2.12)	1.85 (1.60–2.11)	1.83 (1.55–2.09)	1.71 (1.53–2.00)	1.83 (1.54–2.11)	0.11
**Transplant Characteristics**								
CIT Groups								0.51
<8 hours	6,686 (67.9%)	1,794 (68.1%)	718 (65.3%)	490 (65.6%)	138 (62.7%)	92 (62.2%)	97 (69.3%)	
8 to 12 hours	2,718 (27.6%)	727 (27.6%)	333 (30.3%)	220 (29.5%)	71 (32.3%)	50 (33.8%)	39 (27.9%)	
≥12 hours	439 (4.5%)	112 (4.3%)	49 (4.5%)	37 (5.0%)	11 (5.0%)	6 (4.1%)	4 (2.9%)	
**Recipient Characteristics**								
Age, years	55.6 ± 9.1	55.7 ± 8.8	56.3 ± 8.2	55.5 ± 9.5	56.4 ± 8.6	54.2 ± 9.3	54.9 ± 9.3	0.12
Body Mass Index, kg/m^2^	28.7 ± 5.6	28.8 ± 5.6	29.0 ± 5.7	28.9 ± 6.0	28.8 ± 5.7	28.5 ± 5.6	29.0 ± 5.8	0.58
MELD Score	22 (15–29)	22 (15–29)	21 (14–29)	21 (15–28)	21 (15–28)	21 (15–27)	20 (15–27)	0.68
Etiology of ESLD^b^								0.60
Acute Liver Failure	138 (1.4%)	30 (1.1%)	10 (0.9%)	14 (1.8%)	1 (0.5%)	2 (1.3%)	2 (1.4%)	
CC/NASH	1,206 (12.1%)	330 (12.4%)	154 (13.7%)	109 (14.3%)	31 (13.9%)	15 (10.0%)	21 (14.8%)	
Cholestatic Disease	704 (7.0%)	200 (7.5%)	62 (5.5%)	61 (8.0%)	18 (8.1%)	11 (7.3%)	10 (7.0%)	
Cirrhosis (NOS)	410 (4.1%)	116 (4.3%)	48 (4.3%)	26 (3.4%)	9 (4.0%)	7 (4.7%)	9 (6.3%)	
Congenital/Metabolic	305 (3.1%)	56 (2.1%)	27 (2.4%)	15 (2.0%)	2 (0.9%)	5 (3.3%)	3 (2.1%)	
Alcohol	1,262 (12.6%)	357 (13.4%)	150 (13.4%)	104 (13.7%)	32 (14.4%)	25 (16.7%)	18 (12.7%)	
HBV	144 (1.4%)	43 (1.6%)	20 (1.8%)	14 (1.8%)	3 (1.4%)	2 (1.3%)	2 (1.4%)	
HCV	2,795 (28.3%)	735 (27.5%)	301 (26.8%)	190 (24.9%)	51 (22.9%)	42 (28.0%)	38 (26.8%)	
HCC	2,833 (28.3%)	759 (28.4%)	321 (28.6%)	217 (28.5%)	73 (32.7%)	39 (26.0%)	38 (26.8%)	
Other	202 (2.0%)	47 (1.8%)	29 (2.6%)	12 (1.6%)	3 (1.4%)	2 (1.3%)	1 (0.7%)	
Diabetes	2,586 (26.2%)	677 (25.6%)	292 (26.2%)	201 (26.5%)	55 (24.8%)	30 (20.1%)	39 (28.1%)	0.72
Prior TIPS	901 (9.2%)	282 (10.7%)	111 (10.0%)	76 (10.1%)	17 (7.7%)	12 (8.2%)	17 (12.2%)	0.18
PV Thrombosis	1,005 (10.2%)	281 (10.7%)	109 (9.8%)	75 (9.9%)	23 (10.4%)	15 (10.1%)	15 (10.6%)	0.99
Dialysis within 1 week of Transplant	612 (6.1%)	175 (6.6%)	76 (6.8%)	42 (5.5%)	12 (5.4%)	6 (4.0%)	4 (2.8%)	0.40
Ventilator Support	264 (2.6%)	65 (2.4%)	23 (2.1%)	12 (1.6%)	7 (3.1%)	1 (0.7%)	3 (2.1%)	0.32

Graft MaS was significantly associated with graft survival at all time points in multivariable analyses (Table 3, complete multivariable models presented in S3A–S3C Table). Compared to organs with MaS 0–9%, there was an increased likelihood of graft loss at 30 days with MaS levels as low as 10–19%. Allografts with 40–49% and 50–59% MaS carried the highest likelihood of graft loss. Notably, donor age, recipient age, and recipient MELD score were not significantly associated with 30-day graft loss amongst transplants with a donor biopsy. Allografts with 40–49% MaS and 50–59% MaS continued to have an increased likelihood of graft loss at 90-days and 1-year after transplantation. Grafts with lower levels of MaS, however, did not have a significantly elevated likelihood of graft loss after 30 days from transplant. Other factors associated with graft survival are reported in supplemental materials.

**Table 3:** Likelihood of graft loss with donor liver macrosteatosis after transplant.

	Unadjusted			Adjusted^a^		
	**OR**	**95% CI**	**p-Value**	**OR**	**95% CI**	**p-Value**
**30-Day Graft Loss**						
Biopsy Result- % MaS			<0.001			<0.001
0 to 9%	*Reference*			*Reference*		
10 to 19%	1.273	1.041 – 1.557		1.301	1.055 – 1.605	
20 to 29%	1.351	1.020 – 1.789		1.311	0.972 – 1.768	
30 to 39%	1.564	1.142 – 2.142		1.488	1.061 – 2.088	
40 to 49%	2.553	1.610 – 4.047		2.466	1.522 – 3.993	
50 to 59%	2.925	1.724 – 4.960		2.921	1.672 – 5.103	
≥60%	1.645	0.831 – 3.255		1.731	0.862 – 3.477	
**90-Day Graft Loss**						
Biopsy Result- % MaS			<0.001			<0.001
0 to 9%	*Reference*			*Reference*		
10 to 19%	1.144	0.967 – 1.353		1.145	0.961 – 1.364	
20 to 29%	1.277	1.013 – 1.608		1.230	0.962 – 1.573	
30 to 39%	1.360	1.040 – 1.778		1.320	0.992 – 1.755	
40 to 49%	2.150	1.435 – 3.223		2.119	1.390 – 3.231	
50 to 59%	2.155	1.323 – 3.511		2.265	1.356 – 3.786	
≥60%	1.361	0.749 – 2.472		1.391	0.755 – 2.563	
**1-Year Graft Loss**						
Biopsy Result- % MaS			0.09			0.02
0 to 9%	*Reference*			*Reference*		
10 to 19%	0.987	0.866 – 1.123		0.959	0.837 – 1.099	
20 to 29%	1.108	0.924 – 1.329		1.106	0.913 – 1.339	
30 to 39%	1.088	0.876 – 1.352		1.075	0.854 – 1.353	
40 to 49%	1.415	0.982 – 2.039		1.465	1.002 – 2.142	
50 to 59%	1.710	1.311 – 2.586		1.978	1.281 – 3.056	
≥60%	1.179	0.730 – 1.906		1.335	0.814 – 2.189	

^a^Adjusted models present OR [95% CI] derived from multivariable models including factors significantly associated with allograft survival within respective cohorts at each follow-up duration.

### Sub-group analysis in Lower and Higher MELD cohorts

**Fig 2:**
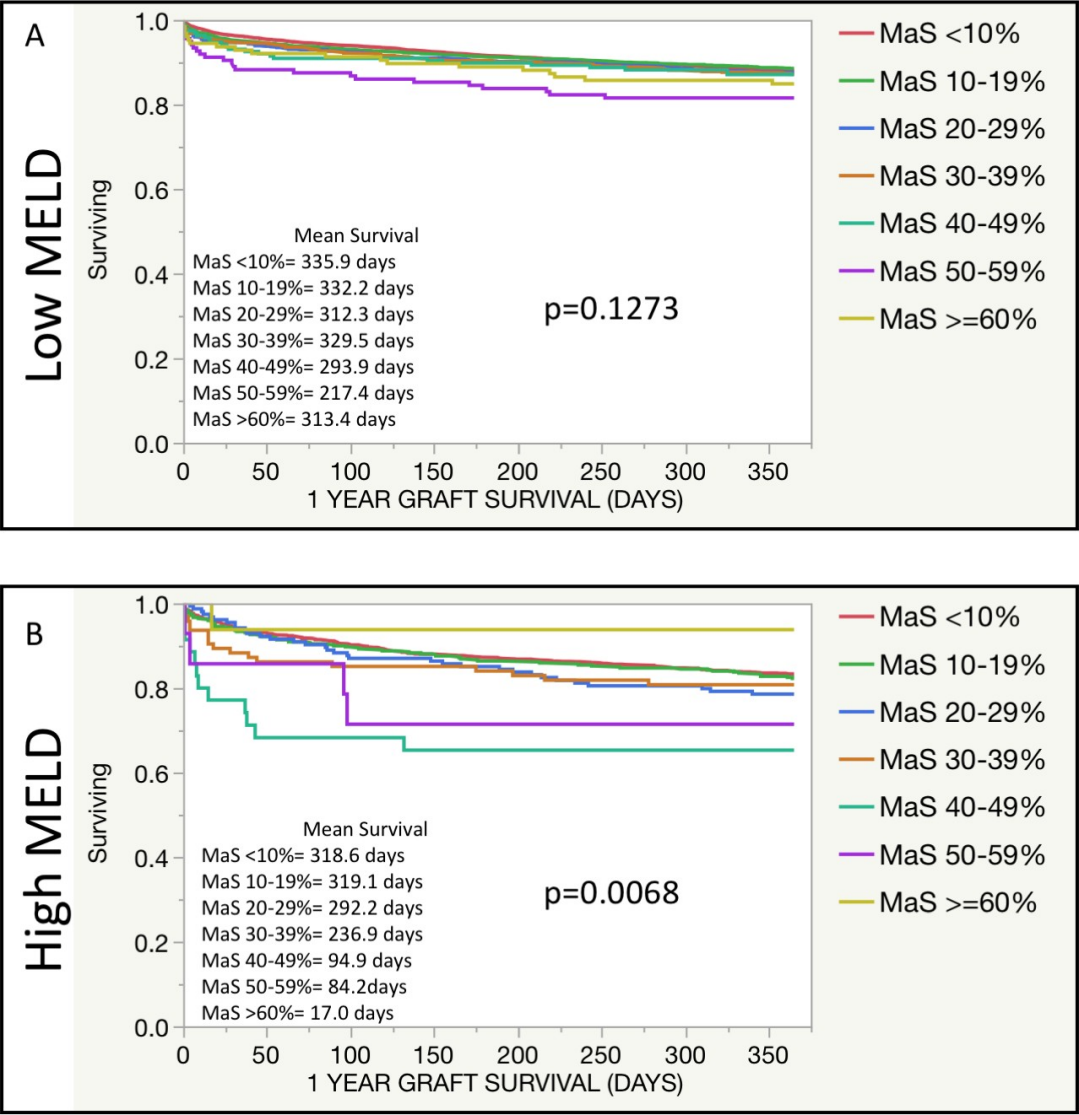
Allograft survival for organs with donor liver biopsy by MELD group. KM survival analyses were performed for 1-year allograft survival in Low MELD (A) and High MELD (B) recipients. Survival was compared across MaS groups. Allograft survival was truncated to 365 days; mean allograft survival is reported.

KM survival analyses showed no significant difference across MaS groups for 1-year graft survival in Lower MELD recipients (Fig 2A, p = 0.12). Conversely, in Higher MELD recipients, there was a significant difference in graft survival by MaS groups (Fig 2B, p = 0.007).

Amongst Lower MELD recipients, allograft MaS was an independent predictor of graft loss at 30-days and 90-days, but not at 1-year after transplant (Table 4, complete models in S4A–S4C Table). At 30 days post-transplant, the likelihood of graft loss was highest for allografts with 50–59% MaS (p = 0.002). At 90 days post-transplant a similar pattern was seen with the highest likelihood of graft loss in organs with 50–59% MaS (p = 0.04). Although allograft MaS was not an independent predictor of graft loss at 1-year, the odds of graft loss was again the highest for organs with 50–59% MaS (p = 0.14).

**Table 4:** Odds of graft loss by MaS groups in Low and High MELD recipients*.

	Lower MELD Recipients			Higher MELD Recipients		
	**OR**	**95% CI**	**p-Value**	**OR**	**95% CI**	**p-Value**
**30-Day Graft Loss**						
Biopsy Result- % MaS			0.002			0.002
0 to 9%	*Reference*			*Reference*		
10 to 19%	1.368	1.085 – 1.725		0.991	0.604 – 1.627	
20 to 29%	1.373	0.993 – 1.898		0.847	0.376 – 1.909	
30 to 39%	1.280	0.869 – 1.885		2.365	1.163 – 4.810	
40 to 49%	1.807	0.984 – 3.319		6.878	2.631 – 17.984	
50 to 59%	2.962	1.627 – 5.391		2.552	0.499 – 13.034	
≥60%	1.715	0.815 – 3.607		1.383	0.168 – 11.394	
**90-Day Graft Loss**						
Biopsy Result- % MaS			0.04			0.002
0 to 9%	*Reference*			*Reference*		
10 to 19%	1.175	0.967 – 1.427		1.017	0.684 – 1.514	
20 to 29%	1.178	0.895 – 1.550		1.337	0.763 – 2.344	
30 to 39%	1.187	0.862 – 1.636		1.846	0.919 – 3.708	
40 to 49%	1.537	0.905 – 2.612		6.687	3.024 – 14.790	
50 to 59%	2.350	1.366 – 4.042		1.659	0.339 – 8.099	
≥60%	1.494	0.788 – 2.831		0.613	0.076 – 4.914	
**1-Year Graft Loss**						
Biopsy Result- % MaS			0.14			0.01
0 to 9%	*Reference*			*Reference*		
10 to 19%	0.950	0.817 – 1.105		1.015	0.745 – 1.383	
20 to 29%	1.032	0.833 – 1.278		1.426	0.923 – 2.201	
30 to 39%	1.037	0.807 – 1.334		1.151	0.656 – 2.019	
40 to 49%	1.150	0.732 – 1.808		3.986	1.841 – 8.634	
50 to 59%	1.871	1.178 – 2.970		2.720	0.756 – 9.785	
≥60%	1.501	0.903 – 2.494		0.355	0.045 – 2.827	

*Adjusted OR presented were derived from multivariable models which adjusted for factors significantly associated with allograft survival for respective cohorts at each follow-up duration.

Allograft MaS was significantly associated with graft loss at all time points for Higher MELD recipients (Table 4, complete models in S5A–S5C Table). At 30 days after transplantation, likelihood of graft loss peaked for organs with 40–49% MaS (p = 0.002). At 90 days after transplant with the highest odds of graft loss was seen amongst allografts with 40–49% MaS (p = 0.002). Importantly, at 90 days post-transplant, the risk of graft loss was not statistically different between organs with 0–9% and 30–39% MaS. At 1-year, likelihood of graft loss did not significantly differ across allografts with <40% MaS. Organs with MaS 40–49% however had persistently increased likelihood of graft loss (p = 0.01).

### Graft loss with and without donor biopsy

As shown above, allografts without a donor biopsy have slightly better graft survival and may represent a higher standard for allograft survival. Therefore, allograft survival for organs without a donor biopsy was compared to those with a biopsy, by degree of MaS seen on biopsy.

**Fig 3:**
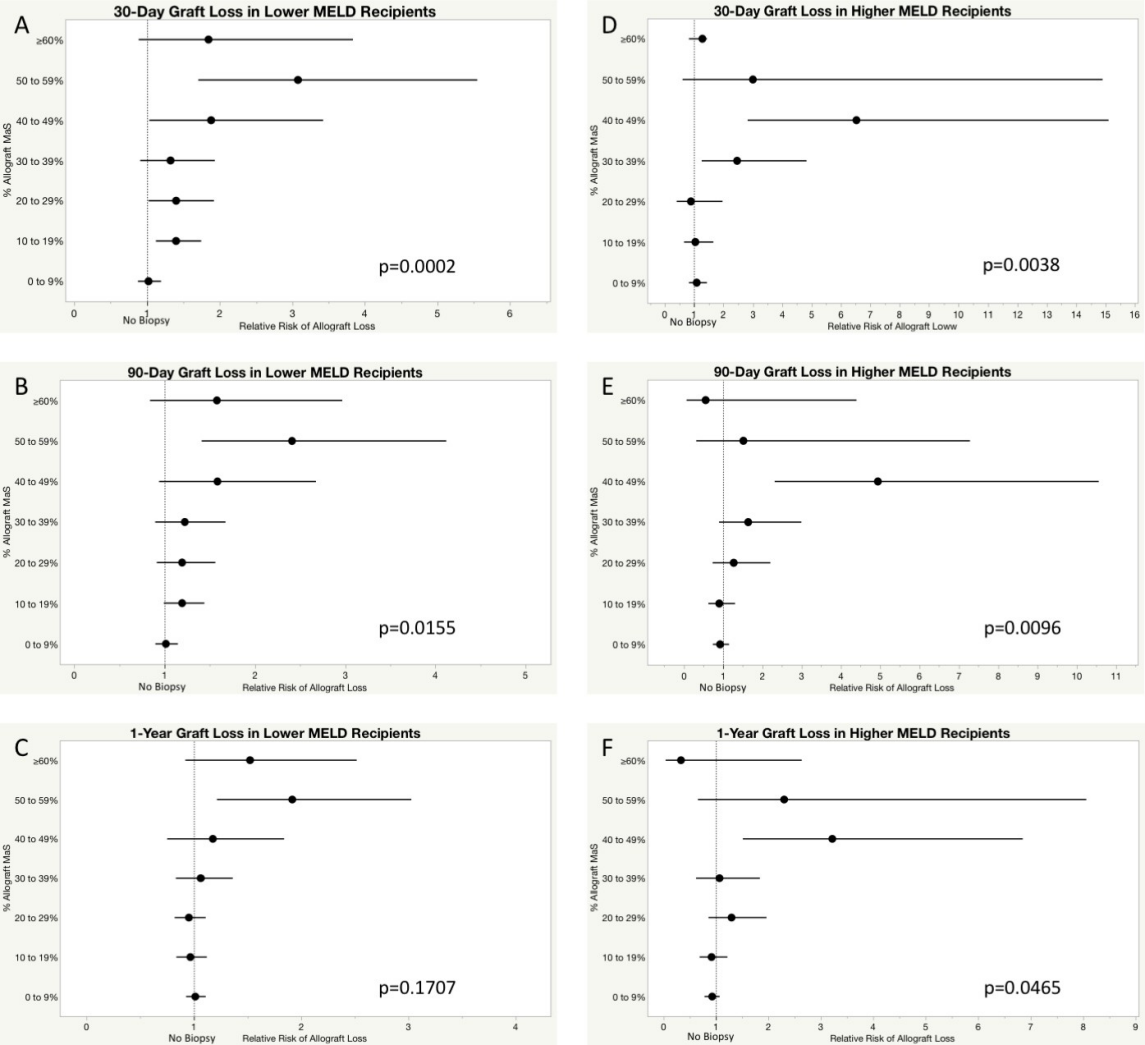
Relative risk of graft loss compared to organs without donor liver biopsy. Forest plot representing the relative risk of allograft loss determined by multivariable logistic regression models. Risk of graft loss in Lower MELD recipients at A) 30 days, B) 90 days, C) 1 year; risk of graft loss in Higher MELD recipients at D) 30 days, E) 90 days, and F) 1 year.

In Lower MELD recipients, MaS level was significantly associated with 30-day and 90-day allograft survival, but again was not significantly associated with 1-year graft survival (Fig 3A–3C). At 30 days post-transplant, there was increased likelihood of graft loss with MaS levels as low as 10–19% compared to No Biopsy recipients (p<0.001). The odds of graft loss peaked for organs with 50–59% MaS. At 90 days, the likelihood of graft loss remained significantly increased for allografts with 50–59% (p = 0.02), while allografts with lower levels of MaS had similar odds of graft loss those without a biopsy. At 1-year post-transplant, there was increased risk of graft loss with organs showing 50–59% MaS, however graft MaS was not significantly associated with graft survival overall (p = 0.17).

In Higher MELD recipients, allograft MaS was associated with graft survival at all time points (Fig 3D–3F). Unlike Lower MELD recipients, however, risk of graft loss at 30 days post-transplant did not significantly increase until MaS levels reached 30–39% (p = 0.004) and was highest for organs with MaS 40–49%. At 90 days, MaS levels of 40–49% were associated with increased likelihood of graft loss compared to No Biopsy cohort (p = 0.009). At 1-year, there remained an increased likelihood of graft loss for organs with MaS 40–49% compared to No Biopsy grafts (p = 0.046).

### Graft survival with elevated MaS thresholds

The likelihood of graft loss across MaS subgroups presented here suggest that Lower MELD recipients may tolerate allografts with up to 50% MaS and Higher MELD recipients may tolerate allografts with up to 40% MaS without significant decrease in graft survival. Therefore, these new MaS thresholds were established and graft survival was evaluated compared to organs transplanted without a pre-donation biopsy.

**Fig 4:**
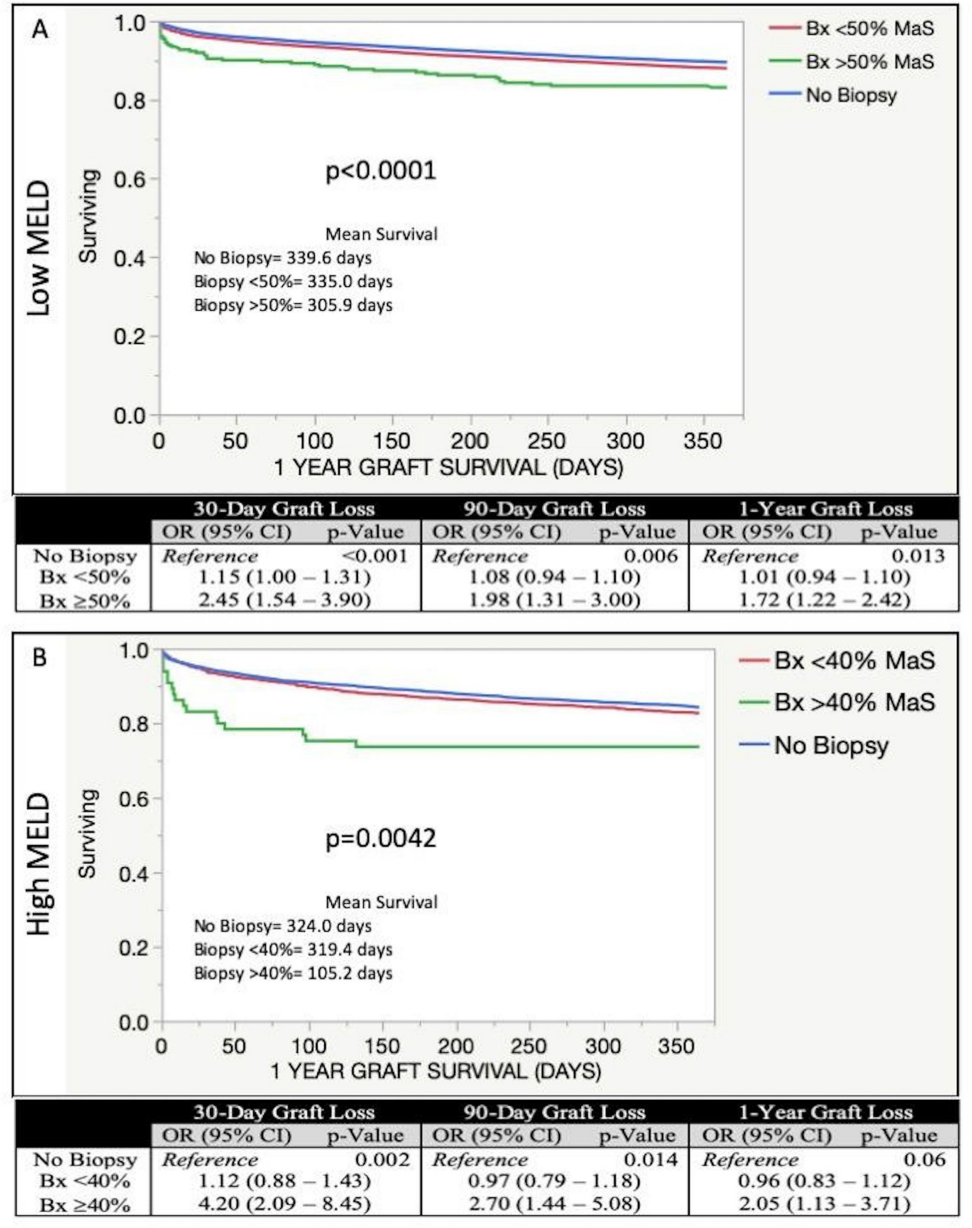
Allograft survival for organs with and without donor liver biopsy. Organs with biopsy are grouped into new MaS cut-off groups. KM Survival analysis is performed to compare organs without a donor biopsy to those with a donor biopsy. Likelihood of graft loss assessed by multivariable logistic regression with odds ratios reported. A) Low MELD recipients comparing those without biopsy to those with a biopsy showing <50% MaS and ≥50% MaS. B) High MELD recipients comparing those without biopsy to those with biopsy showing <40% MaS and ≥40% MaS. Allograft survival truncated to 365 days; mean allograft survival is reported.

KM survival analyses showed significant differences in 1-year graft survival in both Lower MELD (Fig 4A) and Higher MELD (Fig 4B) recipients with thresholds of 50% MaS and 40% MaS, respectively. In Lower MELD recipients, MaS ≥50% carried an increased likelihood of graft loss at 30-days, 90-days and 1-year after transplant. Amongst Higher MELD recipients, organs with MaS ≥40% showed increased odds of graft loss at 30- and 90-days. At 1-year, the odds of graft loss were increased with MaS ≥40%; however, with inclusion of No Biopsy recipients, allograft MaS was not significantly associated with graft survival overall (p = 0.06). Importantly, the likelihood of graft loss for organs below MaS thresholds of 50% in Lower MELD and 40% in Higher MELD recipients was not significantly different than those without a donor biopsy at any time point.

## Discussion

Historically, organs with MaS levels >30% have been deemed unsuitable for transplant due to an increased risk of PNF, EAD, and poor outcomes overall.[8,12] As waitlists for liver transplant continue to grow in the United States, there has been increasing support for the use of marginal organs, including those with increased levels of MaS. Small, single-institution studies have reported successful outcomes with the use of high MaS organs; however, no large cohort has been studied. Therefore, we used a large nationally derived database to evaluate the outcomes of high MaS organs and sought to establish new thresholds for acceptable MaS to increase the donor pool without detriment to outcomes in liver transplantation.

Using the OPTN STAR database, we were able to compile a group of 16,050 transplants from across the United States with information on MaS levels after donor liver biopsy, representing the largest cohort evaluated to date. In an analysis of all transplants with biopsy data, allografts with MaS levels of 10–19% and 20–29% carried an increased likelihood of graft loss at 30-days, compared to those with 0–9% MaS. This finding is important, as it suggests that even low levels of MaS present a risk factor for early graft loss. Furthermore, likelihood of 30-day graft loss more than doubled with MaS 40–49% and showed a nearly threefold increase for allografts with MaS 50–59%.

By convention, organs with 30–60% MaS have been combined into a single “Moderate” steatosis group. Wu et al. recently performed a systematic review and found organs with moderate steatosis had increased risk of PNF but no difference in 1-year mortality.[25] The results of our study show that risk is not evenly distributed across the range of moderate MaS. While we found increased risk of 30-day graft loss for multiple levels of MaS, the likelihood for long-term graft loss, specifically at 1-year after transplant, was elevated with MaS levels of 40–49% and 50–59% but not 30–39%.

The allocation of organs is highly dependent on recipient MELD score. Allocation policies have recently undergone multiple changes since the implementation of a MELD-based system in 2003.[26,27] The most recent policies incorporate both recipient MELD score as well as geographic distribution. The MELD groups identified in this study reflect these allocation policies by establishing a cutoff score of 33 to differentiate Lower and Higher MELD recipients. Prior studies on steatosis have evaluated outcomes in primarily Lower MELD recipients. This study showed that amongst all biopsied organs, recipient MELD was not a significant predictor of 30-day graft loss when other recipient factors (i.e. ventilator dependence, dialysis, and portal vein thrombosis, etc.) were considered. However, recipient MELD score was associated with 90-day and 1-year risks of graft loss.

Subgroup analysis of Lower and Higher MELD recipients receiving organs with MaS revealed different 1-year graft survival on KM analysis. Mean graft survival for an organ with 40–49% MaS in a Lower MELD recipient was 293.9 days, while it was only 94.9 days in a Higher MELD recipient. Similarly, a 50–59% MaS graft had mean survival of 217.4 days in Low MELD recipients and 84.2 days in Higher MELD recipients. Differences existed but were smaller for lower MaS organs. Notably, there were significant decreases in mean graft survival at 50–59% MaS in Low MELD recipients and 40–49% in High MELD recipients. These findings were reflected in multivariable analyses of risk of graft loss. In Low MELD recipients, there was increased odds of graft loss with organs having MaS 50–59% at all time points. High MELD recipients showed an increased likelihood of graft loss with organs over 40% MaS at all time points.

To date, this is the first study to evaluate graft survival for steatotic organs in Low and High MELD recipients separately. These results are important for recipient selection during the organ allocation process. The risk of graft loss with organs showing 30–39% MaS was not significantly different from lower MaS organs in Low MELD recipients, and only increased at 30-days in High MELD recipients. These data again show that organs previously singly grouped as moderate steatosis actually show variable outcomes. Furthermore, it highlights the importance of recipient selection for high MaS organs.

This study showed significantly different 1-year graft survival between organs with and without donor biopsy, therefore, outcomes were assessed against a No Biopsy cohort. The difference in outcomes is likely related to donor quality, as those who remained without a biopsy were younger and had fewer comorbidities. The No Biopsy cohort provides a positive control group to measure outcomes against. This additional comparison provides a frame of reference by which to interpret outcomes. Notably, similar outcomes between organs with and without biopsy suggest these marginal organs are likely to be successful despite differences in other donor characteristics. This study demonstrated that in Lower and Higher MELD recipients, organs with <30% and 30–39% MaS did not have significantly different risks of graft compared to those without a biopsy at 90 days and 1-year post-transplant. Furthremore, this study established new threshold cutoffs for allograft MaS of 50% MaS in Lower MELD and 40% MaS in Higher MELD recipients. KM survival analyses showed significantly different 1-year graft survival between these high MaS organs and the lower MaS and No Biopsy organs in both Lower and Higher MELD populations. Notably, these thresholds are higher than previously proposed 30% MaS.

The results of this study overall suggest that allografts with increased levels of MaS may be transplanted successfully in both Lower and Higher MELD recipients. The implications of these findings would increase the number of potential donors by accepting these organs for transplant. Previously, our group reported that there is a significant association between UNOS Region and acceptance of organs with increased MaS, but overall utilization rates remained low.[28] Further evaluation of donor characteristics amongst discarded organs with higher levels of MaS is indicated to truly determine the potential impact of these findings on the donor pool.

Despite no statistically significant difference in lower MaS organ survival compared to those without a biopsy in both Lower and Higher MELD groups, there remains improved graft survival in lower MELD recipients for high MaS organs. Still, we have found that organs up to 40% MaS may be used without significant detriment in outcomes for High MELD recipients, which is notable as these are the recipients most in need. Unfortunately, outcomes research in transplantation is significantly hindered by selection bias, as outcomes from discarded organs are unknown. Pursuit of prospective studies would be similarly difficult due to outcomes-based accreditation programs which limit the risk programs are willing to undertake with such studies.

This study has many strengths and limitations. There are likely additional factors and interactions which contribute to both positive and poor outcomes with the use highly steatotic organs, which are not recognized and will remain in question due to bias and variation inherent to donor and recipient selection and post-transplant care. Still, as the largest and most comprehensive study to date, the results from this study could potentially provide guidelines to carry out such a study, with threshold MaS values to better evaluate the role of other factors such as donor and recipient ages and comorbidities. Furthermore, this study was the first to evaluate differences in outcomes directly related to recipient MELD to account for recipient variability and found that organs with levels of steatosis up to 50% may be used with good outcomes in Lower MELD recipients.

Additional limitations must be acknowledged in this study. First, this is a retrospective study of prospectively collected data and only a prospective study would provide more definitive results. Additionally, the analysis is limited to the factors provided within the database and studies have shown additional factors to significantly influence outcomes, such as recipient infection or use of vasopressors.[29] Second, only 8.6% of biopsied donors had MaS ≥30%, with very low numbers of organs with MaS 40–49%, 50–59% and ≥60%. Still, the population in this study represents the largest cohort evaluated from a national database to date. With such a large population, we recognize that statistical significance may be present with small differences; however, we have attempted to delineate the clinical significance of these findings. Third, donor liver biopsies may show liver fibrosis or cellular damage, in addition to steatosis, which all may contribute to the decision to reject an organ. The OPTN STAR database however only captures steatosis and therefore we are limited in our ability to comment fully on organ discard practices. Furthermore, prior studies have shown significant inter-observer variability in frozen section interpretation as well as the definitions of macrosteatosis, microsteatosis, and other histologic findings.[30–34] A notable finding throughout this study was consistently better results from organs with MaS ≥60% compared to those with lower levels. This is likely explained by organ selection as these donors were younger with fewer comorbidities. Lastly, a number of important groups were excluded from evaluation, including DCD donors and Status 1 recipients. The influence of organ MaS on outcomes of these organs should be investigated.

As transplant waitlists see a rising number of new registrants each year there remains a persistent disparity between allograft availability and waiting candidates. To bridge this gap, there has been increased importance placed on the use of marginal organs. However, best practices for safe and effective use of these remain in question. Macrosteatosis has long been associated with a threshold of 30% for acceptable risk with organ utilization. Our study shows that in the modern transplant era, allografts with increased MaS levels, up to 50%, may be used safely with appropriate recipient selection. The results of this study support the use of these donors and should encourage the utilization of more donors with higher levels of MaS in both Lower and Higher MELD recipients.

## Supporting information

S1 Table Complete donor and recipient characteristics. Demographic, medical and personal characteristics of all donors and recipients evaluated are presented. Donors with and without liver biopsy are compared.
(DOCX)

S2 Table Donor and recipient characteristics by MaS on donor liver biopsy. Comprehensive evaluation of demographic and medical characteristics of all donors and recipients, categorized by percent MaS on biopsy. MaS groups are compared.
(DOCX)

S3 Table Logistic regression modeling for graft loss amongst all organs with biopsy. Multivariable regression analyses for 30-Day (A), 90-Day (B), and 1-Year (C) graft loss.
(DOCX)

S4 Table Logistic regression modeling for graft loss in lower MELD recipients. Multivariable regression analyses for 30-Day (A), 90-Day (B), and 1-Year (C) graft loss amongst recipients with MELD Score <33. Factors known to influence allograft outcomes forced into models (ie. donor age, recipient age, etiology of ESLD).
(DOCX)

S5 Table Logistic regression modeling for graft loss in Higher MELD recipients. Multivariable regression analyses for 30-Day (A), 90-Day (B), and 1-Year (C) graft loss amongst recipients with MELD Scores 33 and higher. Factors known to influence allograft outcomes forced into models (ie. donor age, recipient age, etiology of ESLD).
(DOCX)

## Abbreviations

AUROC: area under receiver operator curve
BMI: body mass index
CIT: cold ischemia time
CMV: cytomegalovirus
DCD: donation after circulatory (or cardiac) death
HBV: hepatitis B virus
HCV: hepatitis C virus
EAD: early allograft dysfunction
EBV: Epstein-Barr virus
ESLD: end-stage liver disease
LDLT: living donor liver transplant
LT: liver transplant
MaS: macrosteatosis
MELD: model for end-stage liver disease
OPTN: Organ Procurement and Transplant Network
PNF: primary non-function
PV: portal vein
STAR: Standard Transplant Analysis and Research
TIPS: transjugular intrahepatic portovenous shunt

## References

[1] Kim WR, Lake JR, Smith JM, et al OPTN/SRTR 2017 Annual Data Report: Liver. *Am J Transplant*. 2019;19 Suppl 2:184–283. 30811890 10.1111/ajt.15276

[2] Pezzati D, Ghinolfi D, De Simone P, Balzano E, Filipponi F. Strategies to optimize the use of marginal donors in liver transplantation. *World J Hepatol*. 2015;7(26):2636–2647. 10.4254/wjh.v7.i26.2636 26609341 PMC4651908

[3] Verran D, Kusyk T, Painter D, et al Clinical experience gained from the use of 120 steatotic donor livers for orthotopic liver transplantation. *Liver Transpl*. 2003;9(5):500–505. 10.1053/jlts.2003.50099 12740794

[4] Halon A, Patrzalek D, Rabczynski J. Hepatic steatosis in liver transplant donors: rare phenomenon or common feature of donor population? *Transplant Proc*. 2006;38(1):193–195. 10.1016/j.transproceed.2005.11.088 16504700

[5] Estes C, Razavi H, Loomba R, Younossi Z, Sanyal AJ. Modeling the epidemic of nonalcoholic fatty liver disease demonstrates an exponential increase in burden of disease. *Hepatology*. 2018;67(1):123–133. 10.1002/hep.29466 28802062 PMC5767767

[6] Pais R, Barritt ASt, Calmus Y, et al NAFLD and liver transplantation: Current burden and expected challenges. *J Hepatol*. 2016;65(6):1245–1257. 10.1016/j.jhep.2016.07.033 27486010 PMC5326676

[7] Adam R, Reynes M, Johann M, et al The outcome of steatotic grafts in liver transplantation. *Transplant Proc*. 1991;23(1 Pt 2):1538–1540. 1989281

[8] Todo S, Demetris AJ, Makowka L, et al Primary nonfunction of hepatic allografts with preexisting fatty infiltration. *Transplantation*. 1989;47(5):903–905. 10.1097/00007890-198905000-00034 2655230 PMC2967252

[9] Trevisani F, Colantoni A, Caraceni P, Van Thiel DH. The use of donor fatty liver for liver transplantation: a challenge or a quagmire? *J Hepatol*. 1996;24(1):114–121. 10.1016/s0168-8278(96)80195-4 8834034

[10] Andert A, Ulmer TF, Schoning W, et al Grade of donor liver microvesicular steatosis does not affect the postoperative outcome after liver transplantation. *Hepatobiliary Pancreat Dis Int*. 2017;16(6):617–623. 10.1016/S1499-3872(17)60064-X 29291781

[11] Kwon CH, Joh JW, Lee KW, et al Safety of donors with fatty liver in liver transplantation. *Transplant Proc*. 2006;38(7):2106–2107. 10.1016/j.transproceed.2006.07.018 16980014

[12] Baccarani U, Adani GL, Isola M, et al Steatosis of the graft is a risk factor for posttransplantation biliary complications. *Transplant Proc*. 2009;41(4):1313–1315. 10.1016/j.transproceed.2009.03.084 19460549

[13] Spitzer AL, Lao OB, Dick AA, et al The biopsied donor liver: incorporating macrosteatosis into high-risk donor assessment. *Liver Transpl*. 2010;16(7):874–884. 10.1002/lt.22085 20583086

[14] McCormack L, Petrowsky H, Jochum W, Mullhaupt B, Weber M, Clavien PA. Use of severely steatotic grafts in liver transplantation: a matched case-control study. *Ann Surg*. 2007;246(6):940–946; discussion 946–948. 10.1097/SLA.0b013e31815c2a3f 18043095

[15] Deroose JP, Kazemier G, Zondervan P, Ijzermans JN, Metselaar HJ, Alwayn IP. Hepatic steatosis is not always a contraindication for cadaveric liver transplantation. *HPB (Oxford)*. 2011;13(6):417–425. 21609375 10.1111/j.1477-2574.2011.00310.x PMC3103099

[16] Doyle MB, Vachharajani N, Wellen JR, et al Short- and long-term outcomes after steatotic liver transplantation. *Arch Surg*. 2010;145(7):653–660. 10.1001/archsurg.2010.119 20644128

[17] Angele MK, Rentsch M, Hartl WH, et al Effect of graft steatosis on liver function and organ survival after liver transplantation. *Am J Surg*. 2008;195(2):214–220. 10.1016/j.amjsurg.2007.02.023 18154767

[18] Li J, Liu B, Yan LN, et al Reversal of graft steatosis after liver transplantation: prospective study. *Transplant Proc*. 2009;41(9):3560–3563. 10.1016/j.transproceed.2009.06.222 19917344

[19] Chavin KD, Taber DJ, Norcross M, et al Safe use of highly steatotic livers by utilizing a donor/recipient clinical algorithm. *Clin Transplant*. 2013;27(5):732–741. 10.1111/ctr.12211 23991646

[20] Gabrielli M, Moisan F, Vidal M, et al Steatotic livers. Can we use them in OLTX? Outcome data from a prospective baseline liver biopsy study. *Ann Hepatol*. 2012;11(6):891–898. 23109453

[21] de Graaf EL, Kench J, Dilworth P, et al Grade of deceased donor liver macrovesicular steatosis impacts graft and recipient outcomes more than the Donor Risk Index. *J Gastroenterol Hepatol*. 2012;27(3):540–546. 10.1111/j.1440-1746.2011.06844.x 21777274

[22] Noujaim HM, de Ville de Goyet J, Montero EF, et al Expanding postmortem donor pool using steatotic liver grafts: a new look. *Transplantation*. 2009;87(6):919–925. 10.1097/TP.0b013e31819b3f76 19300197

[23] Wong TC, Fung JY, Chok KS, et al Excellent outcomes of liver transplantation using severely steatotic grafts from brain-dead donors. *Liver Transpl*. 2016;22(2):226–236. 10.1002/lt.24335 26359934

[24] Collett D. *Modelling survival data in medical research*. Third edition ed. Boca Raton: CRC Press, Taylor & Francis Group; 2015.

[25] Wu C, Lu C, Xu C. Short-term and long-term outcomes of liver transplantation using moderately and severely steatotic donor livers: A systematic review. *Medicine (Baltimore)*. 2018;97(35):e12026. 30170411 10.1097/MD.0000000000012026 PMC6393101

[26] Kamath PS, Wiesner RH, Malinchoc M, et al A model to predict survival in patients with end-stage liver disease. *Hepatology*. 2001;33(2):464–470. 10.1053/jhep.2001.22172 11172350

[27] Wiesner RH, McDiarmid SV, Kamath PS, et al MELD and PELD: application of survival models to liver allocation. *Liver Transpl*. 2001;7(7):567–580. 10.1053/jlts.2001.25879 11460223

[28] Steggerda JA, Kim IK, Malinoski D, Klein AS, Bloom MB. Regional Variation in Utilization and Outcomes of Liver Allografts From Donors With High Body Mass Index and Graft Macrosteatosis: A Role for Liver Biopsy. *Transplantation*. 2019;103(1):122–130. 10.1097/TP.0000000000002379 30048394

[29] Petrowsky H, Rana A, Kaldas FM, et al Liver transplantation in highest acuity recipients: identifying factors to avoid futility. *Ann Surg*. 2014;259(6):1186–1194. 10.1097/SLA.0000000000000265 24263317

[30] El-Badry AM, Breitenstein S, Jochum W, et al Assessment of hepatic steatosis by expert pathologists: the end of a gold standard. *Ann Surg*. 2009;250(5):691–697. 10.1097/SLA.0b013e3181bcd6dd 19806055

[31] Crowley H, Lewis WD, Gordon F, Jenkins R, Khettry U. Steatosis in donor and transplant liver biopsies. *Hum Pathol*. 2000;31(10):1209–1213. 10.1053/hupa.2000.18473 11070113

[32] Yersiz H, Lee C, Kaldas FM, et al Assessment of hepatic steatosis by transplant surgeon and expert pathologist: a prospective, double-blind evaluation of 201 donor livers. *Liver Transpl*. 2013;19(4):437–449. 10.1002/lt.23615 23408461

[33] Rey JW, Wirges U, Dienes HP, Fries JW. Hepatic steatosis in organ donors: disparity between surgery and histology? *Transplant Proc*. 2009;41(6):2557–2560. 10.1016/j.transproceed.2009.06.121 19715973

[34] Neuberger J. Transplantation: Assessment of liver allograft steatosis. *Nat Rev Gastroenterol Hepatol*. 2013;10(6):328–329. 10.1038/nrgastro.2013.74 23629606

