# Supplementary material for: Higher thresholds for the utilization of steatotic allografts in liver transplantation: Analysis from a U.S. national database
Source: PLoS One. 2020 Apr 2;15(4):e0230995. doi: 10.1371/journal.pone.0230995 (PMC7117730; doi:10.1371/journal.pone.0230995)
Supplement: S1 Table — Demographic, medical and personal characteristics of all donors and recipients evaluated are presented. Donors with and without liver biopsy are compared. (DOCX) [file pone.0230995.s001.docx]

Table S1. Complete Donor and Recipient Characteristics

|  | All Transplants  (n=41,347) | Pre-Donation Biopsy  (n=16,306) | No Biopsy  (n=25,041) | p-Value |
| --- | --- | --- | --- | --- |
| Recipient Characteristics | | | | |
| Age, years (mean±SD, median (IQR)) | 43.0 ± 16.8  44 (28 – 56) | 50.0 ± 14.9  51 (40 – 61) | 38.4 ± 16.3  38 (24 – 51) | <0.001 |
| Age Groups (n, %) |  |  |  | <0.001 |
| <35 years | 14,161 (34.3%) | 2,752 (16.9%) | 11,409 (45.6%) |  |
| 35 to 44 years | 6,524 (15.8%) | 2,490 (15.3%) | 4,304 (16.1%) |  |
| 45 to 54 years | 9,224 (22.3%) | 4,384 (26.9%) | 4,840 (19.3%) |  |
| 55 to 64 years | 7,214 (17.5%) | 3,952 (24.2%) | 3,262 (13.0%) |  |
| ≥65 years | 4,224 (10.2%) | 2,728 (16.7%) | 1,496 (6.0%) |  |
| Gender (female) | 16,891 (40.9%) | 7,555 (46.3%) | 9,336 (37.3%) | <0.001 |
| Ethnicity |  |  |  | <0.001 |
| White | 27,187 (65.8%) | 11,080 (68.0%) | 16,107 (64.3%) |  |
| Black | 7,555 (18.3%) | 3,017 (18.5%) | 4,538 (18.1%) |  |
| Hispanic | 5,047 (12.2%) | 1,591 (9.8%) | 3,456 (13.8%) |  |
| Asian | 1,024 (2.5%) | 397 (2.4%) | 627 (2.5%) |  |
| Other | 534 (1.3%) | 221 (1.4%) | 313 (1.3%) |  |
| Body Mass Index, kg/m^2^ | 27.7 ± 6.3  26.6 (23.3 – 30.8) | 29.5 ± 7.3  28.2 (24.3 – 33.5) | 26.4 ± 5.3  25.8 (22.8 – 29.3) | <0.001 |
| BMI ≥30 kg/m^2^ | 11,902 (28.8%) | 6,543 (40.2%) | 5,359 (21.4%) | <0.001 |
| Blood Type |  |  |  | <0.001 |
| O | 19,061 (46.1%) | 7,714 (47.3%) | 11,347 (45.3%) |  |
| A | 15,523 (37.5%) | 6,144 (37.7%) | 9,379 (37.5%) |  |
| B | 5,277 (12.8%) | 1,949 (12.0%) | 3,328 (13.3%) |  |
| AB | 1,486 (3.6%) | 499 (3.1%) | 987 (3.9%) |  |
| Biopsy Results |  |  |  | N/A |
| 0 to 9% | Biopsy Only | 9,999 (66.4%) | - |  |
| 10 to 19% | - | 2,672 (17.7%) | - |  |
| 20 to 29% | - | 1,122 (7.5%) | - |  |
| 30 to 39% | - | 762 (5.1%) | - |  |
| 40 to 49% | - | 223 (1.5%) | - |  |
| 50 to 59% | - | 150 (1.0%) | - |  |
| ≥60% | - | 142 (0.9%) | - |  |
| Cause of Death |  |  |  | <0.001 |
| Anoxia | 9,566 (23.1%) | 4,225 (25.9%) | 5,341 (21.3%) |  |
| Trauma | 13,622 (33.0%) | 3,537 (21.7%) | 10,085 (40.3%) |  |
| CVA | 17,142 (41.5%) | 8,164 (50.1%) | 8,978 (35.9%) |  |
| Other | 1,017 (2.5%) | 380 (2.3%) | 637 (2.5%) |  |
| Diabetes | 5,153 (12.5%) | 3,159 (19.5%) | 1,994 (8.0%) | <0.001 |
| Hypertension | 15,663 (38.1%) | 8,448 (52.2%) | 7,215 (29.0%) | <0.001 |
| Prior Malignancy | 1,545 (3.8%) | 814 (5.0%) | 731 (2.9%) | <0.001 |
| Prior MI | 1,656 (4.0%) | 979 (6.0%) | 677 (2.7%) | <0.001 |
| Cardiac Arrest Prior to Donation | 3,004 (7.3%) | 1,211 (7.4%) | 1,793 (7.2%) | 0.31 |
| CDC High Risk | 5,103 (12.3%) | 2,051 (12.6%) | 3,052 (12.2%) | 0.24 |
| Cigarette Smoker | 11,129 (26.9%) | 5,587 (34.3%) | 5,542 (22.1%) | <0.001 |
| Any Drug Use | 15,524 (37.6%) | 5,705 (35.0%) | 9,819 (37.6%) | <0.001 |
| HCV-Positive | 1,747 (4.2%) | 1,366 (8.4%) | 381 (1.5%) | <0.001 |
| HBV-Positive | 2,396 (5.8%) | 1,485 (9.1%) | 911 (3.6%) | <0.001 |
| EBV-Positive | 34,447 (83.3%) | 14,037 (86.1%) | 20,410 (81.5%) | <0.001 |
| CMV-Positive | 27,302 (66.0%) | 11,092 (68.0%) | 16,210 (64.7%) | <0.001 |
| Serum Creatinine | 1.1 (0.8 – 1.6) | 1.2 (0.8 – 1.9) | 1.1 (0.8 – 1.5) | <0.001 |
| Serum AST | 43 (26 – 81) | 42 (25 – 81) | 43 (26 – 81) | <0.001 |
| Serum ALT | 34 (21 – 65) | 34 (21 – 66) | 34 (21 – 64) | 0.92 |
| Serum Total Bilirubin | 0.7 (0.5 – 1.1) | 0.7 (0.4 – 1.1) | 0.7 (0.5 – 1.1) | <0.001 |
| Hematocrit | 30.4 (27 – 34.5) | 30.7 (27.2 – 35.0) | 30.2 (27.0 – 34.1) | <0.001 |
|  | | | | |
| Cold Ischemic Time (hours) | 6.7 ± 3.0  6.3 (5.0 – 8.0) | 7.0 ± 2.9  6.6 (5.1 – 8.4) | 6.5 ± 3.0  6.0 (4.8 – 7.9) | <0.001 |
| CIT Groups |  |  |  | <0.001 |
| <8 hours | 29,325 (72.3%) | 10,839 (67.6%) | 18,486 (75.4%) |  |
| 8 to 12 hours | 9,676 (23.9%) | 4,484 (28.0%) | 5,192 (21.2%) |  |
| ≥12 hours | 1,560 (3.9%) | 712 (4.4%) | 848 (3.5%) |  |
|  |  |  |  |  |
| Recipient Characteristics | | | | |
| Age | 55.2 ± 9.4  56 (51 – 61) | 55.7 ± 9.0  57 (51 – 62) | 54.9 ± 9.7  56 (50 – 61) | <0.001 |
| Age Groups |  |  |  | <0.001 |
| <35 years | 1,595 (3.9%) | 505 (3.1%) | 1,090 (4.4%) |  |
| 35 to 44 years | 3,014 (7.3%) | 1,090 (6.7%) | 1,924 (7.7%) |  |
| 45 to 54 years | 12,224 (29.6%) | 4,761 (29.2%) | 7,463 (29.8%) |  |
| 55 to 64 years | 18,737 (45.3%) | 7,578 (46.5%) | 11,159 (44.6%) |  |
| ≥65 years | 5,777 (14.0%) | 2,372 (14.6%) | 3,405 (13.6%) |  |
| Gender (female) | 12,722 (30.8%) | 4,719 (28.9%) | 8,003 (32.0%) | <0.001 |
| Ethnicity |  |  |  | <0.001 |
| White | 29,686 (71.8%) | 12,091 (74.2%) | 17,595 (70.3%) |  |
| Black | 3,763 (9.1%) | 1,466 (9.0%) | 2,297 (9.2%) |  |
| Hispanic | 5,508 (13.3%) | 1,849 (11.3%) | 3,659 (14.6%) |  |
| Asian | 1,883 (4.6%) | 672 (4.1%) | 1,211 (4.8%) |  |
| Other | 507 (1.2%) | 228 (1.4%) | 279 (1.1%) |  |
| Body Mass Index | 28.6 ± 5.7  28.0 (24.5 – 32.2) | 28.8 ± 5.7  28.1 (24.7 – 32.3) | 28.5 ± 5.7  27.9 (24.4 – 32.0) | <0.001 |
| BMI ≥30 kg/m^2^ | 15,242 (36.9%) | 6,204 (38.1%) | 9,038 (36.1%) | <0.001 |
| Blood Type |  |  |  | <0.001 |
| O | 18,047 (44.5%) | 7,489 (45.9%) | 10,918 (43.6%) |  |
| A | 15,061 (36.4%) | 5,985 (36.7%) | 9,076 (36.2%) |  |
| B | 5,688 (13.8%) | 2,095 (12.9%) | 3,593 (14.4%) |  |
| AB | 2,191 (5.3%) | 737 (4.5%) | 1,454 (5.8%) |  |
| MELD Score^a^ | 23.2 ± 10.0  22 (15 – 30) | 22.3 ± 9.5  21 (15 – 29) | 21.3 ± 10.2  23 (16 – 31) | <0.001 |
| MELD Groups |  |  |  | <0.001 |
| Low MELD  (Score <33) | 33,586 (81.3%) | 13,840 (85.0%) | 19,746 (78.9%) |  |
| High MELD  (Score 33-40) | 7,710 (18.7%) | 2,441 (15.0%) | 5,269 (21.1%) |  |
| Exceptions Given |  |  |  | <0.001 |
| No Exception | 25,914 (62.3%) | 9,975 (61.2%) | 15,939 (63.7%) |  |
| HCC Exception | 11,324 (27.4%) | 4,677 (28.7%) | 6,647 (26.5%) |  |
| Other Exception | 4,109 (9.9%) | 1,654 (10.1%) | 2,455 (9.8%) |  |
| Etiology of ESLD^b^ |  |  |  | <0.001 |
| Acute Liver Failure | 687 (1.7%) | 212 (1.3%) | 475 (1.9%) |  |
| CC/NASH | 5,237 (12.7%) | 2,032 (12.5%) | 3,205 (12.8%) |  |
| Cholestatic Disease | 3,177 (7.7%) | 1,157 (7.1%) | 2,020 (8.1%) |  |
| Cirrhosis (NOS) | 1,842 (4.5%) | 678 (4.2%) | 1,164 (4.7%) |  |
| Congenital/Metabolic | 1,120 (2.7%) | 438 (2.7%) | 682 (2.7%) |  |
| Alcohol | 5,139 (12.4%) | 2,112 (13.0%) | 3,027 (12.1%) |  |
| HBV | 738 (1.8%) | 246 (1.5%) | 492 (2.0%) |  |
| HCV | 11,535 (27.9%) | 4,504 (27.6%) | 7,031 (28.1%) |  |
| HCC | 11,037 (26.8%) | 4,604 (28.2%) | 6,469 (25.8%) |  |
| Other | 799 (1.9%) | 323 (2.0%) | 476 (1.9%) |  |
| Diabetes | 10,385 (25.4%) | 4,199 (26.0%) | 6,186 (24.9%) | 0.01 |
| Prior Malignancy | 7,757 (18.8%) | 3,122 (19.2%) | 4,635 (18.5%) | 0.11 |
| EBV-Positive | 25,305 (61.2%) | 10,280 (63.0%) | 15,025 (60.0%) | <0.001 |
| CMV-Positive | 25,543 (67.4%) | 9,788 (65.9%) | 15,755 (68.3%) | <0.001 |
| Prior Abdominal Surgery | 17,443 (43.0%) | 7,001 (43.7%) | 10,442 (42.5%) | 0.02 |
| Prior TIPS | 3,773 (9.3%) | 1,519 (9.5%) | 2,254 (9.1%) | 0.29 |
| PV Thrombosis | 4,160 (10.2%) | 1,625 (10.1%) | 2,535 (10.3%) | 0.57 |
| Encephalopathy | 25,757 (62.3%) | 9,873 (60.6%) | 15,884 (63.4%) | <0.001 |
| Ascites | 31,251 (75.6%) | 12,096 (74.2%) | 19,155 (76.5%) | <0.001 |
| Dialysis within 1 week of Transplant | 3,372 (8.2%) | 1,018 (6.3%) | 2,354 (9.4%) | <0.001 |
| Ventilator Support at Transplant | 1,425 (3.5%) | 410 (2.5%) | 1,015 (4.1%) | <0.001 |
|  |  |  |  |  |
| Region of Transplant |  |  |  | <0.001 |
| 1 | 1,399 (3.4%) | 823 (5.1%) | 576 (2.3%) |  |
| 2 | 4,766 (11.5%) | 2,199 (13.5%) | 2,567 (10.3%) |  |
| 3 | 7,102 (17.2%) | 2,046 (12.6%) | 5,056 (20.2%) |  |
| 4 | 3,983 (9.6%) | 1,292 (7.9%) | 2,691 (10.8%) |  |
| 5 | 5,712 (13.8%) | 1,663 (10.2%) | 4,049 (16.2%) |  |
| 6 | 1,276 (3.1%) | 550 (3.4%) | 726 (2.9%) |  |
| 7 | 3,050 (7.4%) | 1,622 (10.0%) | 1,428 (5.7%) |  |
| 8 | 2,951 (7.1%) | 1,429 (8.8%) | 1,522 (6.1%) |  |
| 9 | 2,610 (6.3%) | 1,333 (8.2%) | 1,277 (5.1%) |  |
| 10 | 3,728 (9.0%) | 1,092 (6.7%) | 2,636 (10.5%) |  |
| 11 | 4,770 (11.5%) | 2,257 (13.8%) | 2,513 (11.5%) |  |
|  |  |  |  |  |
| a- Lab MELD score calculated  b- primary listed diagnosis  Abbreviations: ALT- alanine aminotransferase; AST- asparate aminotransferase; BMI- body mass index; CC- cryptogenic cirrhosis; CDC- Center for Disease Control; CMV- cytomegalovirus; EBV- Epstein-Barr virus; MI- myocardial infarction; Na-MELD- Sodium-MELD; NASH- non-alcoholic steatohepatitis; HBV- Hepatitis B virus; HCC- hepatocellular cancer; HCV- Hepatitis C virus; NOS- not otherwise specificed; PV- portal vein; TIPS- transjugular intrahepatic portovenous shunt | | | | |
