# Supplementary material for: Higher thresholds for the utilization of steatotic allografts in liver transplantation: Analysis from a U.S. national database
Source: PLoS One. 2020 Apr 2;15(4):e0230995. doi: 10.1371/journal.pone.0230995 (PMC7117730; doi:10.1371/journal.pone.0230995)
Supplement: S2 Table — Comprehensive evaluation of demographic and medical characteristics of all donors and recipients, categorized by percent MaS on biopsy. MaS groups are compared. (DOCX) [file pone.0230995.s002.docx]

S2 Table. Donor and Recipient Characteristics by MaS Group on Donor Biopsy

|  | 0-9% MaS  (n=9,999) | 10-19% MaS  (n=2,673) | 20-29% MaS  (n=1,122) | 30-39% MaS  (n=762) | 40-49% MaS  (n=223) | 50-59% MaS  (n=150) | ≥60% MaS  (n=142) | p-Value |
| --- | --- | --- | --- | --- | --- | --- | --- | --- |
| Age, years (mean±SD, median (IQR)) | 50.0 ± 15.4  51 (40 – 61) | 51.5 ± 13.9  53 (43 – 61) | 50.2 ± 13.1  51 (43 – 59) | 50.1 ± 13.1  51 (43 – 60) | 46.2 ± 13.6  48 (37 – 56) | 43.5 ± 12.2  44 (33 – 51) | 46.0 ± 14.2  48 (36 – 56) | <0.001 |
| Age Groups (n, %) |  |  |  |  |  |  |  | <0.001 |
| <35 years | 1,787 (17.9%) | 342 (12.8%) | 152 (13.6%) | 102 (13.4%) | 46 (20.6%) | 42 (28.0%) | 32 (22.5%) |  |
| 35 to 44 years | 1,489 (14.9%) | 400 (15.0%) | 182 (16.2%) | 122 (16.0%) | 51 (22.8%) | 36 (24.0%) | 23 (!6.2%) |  |
| 45 to 54 years | 2,576 (25.8%) | 757 (28.3%) | 358 (31.9%) | 242 (31.8%) | 58 (26.0%) | 41 (27.3%) | 43 (30.3%) |  |
| 55 to 64 years | 2,361 (23.6%) | 702 (26.3%) | 291 (25.0%) | 201 (26.4%) | 53 (23.8%) | 25 (16.7%) | 33 (23.2%) |  |
| ≥65 years | 1,786 (17.9%) | 472 (17.7%) | 149 (13.3%) | 95 (12.5%) | 15 (6.7%) | 6 (4.0%) | 11 (7.8%) |  |
| Gender (female) | 4,661 (46.6%) | 1,264 (47.3%) | 490 (43.7%) | 339 (44.5%) | 88 (39.5%) | 73 (48.7%) | 71 (50.0%) | 0.09 |
| Ethnicity |  |  |  |  |  |  |  | <0.001 |
| White | 6,751 (67.5%) | 1,843 (69.0%) | 781 (69.6%) | 535 (70.2%) | 152 (68.2%) | 102 (68.0%) | 104 (73.2%) |  |
| Black | 2,055 (20.6%) | 404 (15.1%) | 149 (13.3%) | 104 (13.7%) | 17 (7.6%) | 14 (9.3%) | 23 (16.2%) |  |
| Hispanic | 826 (8.3%) | 311 (11.6%) | 136 (12.1%) | 93 (12.2%) | 48 (21.5%) | 30 (20.0%) | 12 (8.5%) |  |
| Asian | 237 (2.4%) | 77 (2.9%) | 32 (2.9%) | 20 (2.6%) | 2 (0.9%) | 3 (2.0%) | 2 (1.4%) |  |
| Other | 130 (1.3%) | 38 (1.4%) | 24 (2.1%) | 10 (1.3%) | 4 (1.8%) | 1 (0.7%) | 1 (0.7%) |  |
| Body Mass Index, kg/m^2^ | 28.7 ± 7.2  27.3 (23.7 – 32.5) | 31.0 ± 7.3  29.9 (25.8 – 35.1) | 31.9 ± 7.2  31.1 (27.1 – 35.7) | 32.1 ± 7.4  31.0 (26.9 – 36.3) | 31.7 ± 7.1  31.4 (26.1 – 36.2) | 30.7 ± 7.4  29.0 (25.1 – 35.3) | 30.8 ± 7.2  30.2 (25.1 – 35.8) | <0.001 |
| BMI ≥30 kg/m^2^ | 3,503 (35.1%) | 1,314 (49.2%) | 626 (55.8%) | 443 (58.3%) | 124 (56.1%) | 64 (42.7%) | 74 (52.1%) | <0.001 |
| Blood Type |  |  |  |  |  |  |  | 0.04 |
| O | 4,728 (47.3%) | 1,236 (46.2%) | 522 (46.5%) | 397 (52.1%) | 102 (45.7%) | 75 (50.0%) | 74 (52.1%) |  |
| A | 3,736 (37.4%) | 1,015 (38.0%) | 441 (39.3%) | 275 (36.1%) | 93 (41.7%) | 55 (36.7%) | 51 (35.9%) |  |
| B | 1,196 (12.0%) | 356 (13.3%) | 131 (11.7%) | 75 (9.8%) | 25 (11.2%) | 16 (10.7%) | 15 (10.6%) |  |
| AB | 339 (3.4%) | 66 (2.5%) | 28 (2.5%) | 15 (2.0%) | 3 (1.4%) | 4 (2.7%) | 2 (1.4%) |  |
| Cause of Death |  |  |  |  |  |  |  | 0.05 |
| Anoxia | 2,634 (26.3%) | 676 (25.3%) | 296 (26.4%) | 178 (23.4%) | 58 (26.0%) | 39 (26.0%) | 40 (28.2%) |  |
| Trauma | 2,159 (21.6%) | 569 (21.3%) | 238 (21.2%) | 172 (22.6%) | 61 (27.4%) | 42 (28.0%) | 32 (22.5%) |  |
| CVA | 4,970 (49.7%) | 1,369 (51.2%) | 569 (50.7%) | 397 (52.1%) | 95 (42.6%) | 66 (44.0%) | 62 (43.7%) |  |
| Other | 236 (2.4%) | 59 (2.2%) | 19 (1.7%) | 15 (2.0%) | 9 (4.0%) | 3 (2.0%) | 8 (5.6%) |  |
| Diabetes | 1,911 (19.2%) | 569 (21.4%) | 219 (19.7%) | 160 (21.1%) | 41 (18.6%) | 17 (11.5%) | 20 (14.3%) | 0.01 |
| Hypertension | 5,115 (51.5%) | 1,434 (54.0%) | 611 (55.2%) | 441 (58.3%) | 98 (44.3%) | 58 (39.5%) | 63 (45.0%) | <0.001 |
| Prior Malignancy | 512 (5.2%) | 152 (5.7%) | 53 (4.8%) | 35 (4.6%) | 7 (3.2%) | 6 (4.0%) | 1 (0.7%) | 0.11 |
| Prior MI | 630 (6.3%) | 152 (5.7%) | 58 (5.2%) | 32 (4.2%) | 13 (5.8%) | 4 (2.7%) | 16 (11.3%) | 0.007 |
| Cardiac Arrest Prior to Donation | 701 (7.0%) | 214 (8.0%) | 72 (6.4%) | 50 (6.6%) | 22 (9.9%) | 11 (7.3%) | 35 (24.7%) | <0.001 |
| CDC High Risk | 1,370 (13.7%) | 293 (11.0%) | 104 (9.3%) | 64 (8.4%) | 26 (11.7%) | 21 (14.0%) | 22 (15.5%) | <0.001 |
| Cigarette Smoker | 3,402 (34.0%) | 955 (35.7%) | 384 (34.2%) | 264 (34.7%) | 73 (32.7%) | 44 (29.3%) | 45 (31.7%) | 0.53 |
| Any Drug Use | 3,537 (35.4%) | 900 (33.7%) | 369 (32.9%) | 258 (33.9%) | 91 (40.8%) | 66 (44.0%) | 58 (40.9%) | 0.01 |
| HCV-Positive | 959 (9.6%) | 172 (6.4%) | 66 (5.9%) | 25 (3.3%) | 6 (2.7%) | 13 (8.7%) | 4 (2.8%) | <0.001 |
| HBV-Positive | 990 (9.9%) | 221 (8.3%) | 88 (7.8%) | 48 (6.3%) | 14 (6.3%) | 7 (4.7%) | 13 (9.2%) | <0.001 |
| EBV-Positive | 8,562 (85.6%) | 2,348 (87.8%) | 1,000 (89.1%) | 651 (85.4%) | 190 (85.2%) | 131 (87.3%) | 123 (86.6%) | 0.007 |
| CMV-Positive | 6,962 (69.3%) | 1,813 (67.8%) | 731 (65.2%) | 485 (63.7%) | 140 (62.8%) | 92 (61.3%) | 87 (61.3%) | <0.001 |
| Serum Creatinine | 1.2 (0.8 – 1.9) | 1.2 (0.9 – 1.8) | 1.1 (0.8 – 1.7) | 1.2 (0.9 – 1.7) | 1.2 (0.8 – 1.8) | 1.1 (0.9 – 1.7) | 1.1 (0.8 – 1.9) | 0.04 |
| Serum AST | 41 (25 – 81) | 41 (25 – 83) | 43 (26 – 79) | 41 (25 – 76) | 44 (24 – 101) | 45 (28 – 84) | 47 (27 – 73) | 0.62 |
| Serum ALT | 33 (20 – 66) | 35 (22 – 70) | 37 (23 – 68) | 36 (22 – 64) | 43 (23 – 77) | 40 (25 – 74) | 42 (23 – 73) | <0.001 |
| Serum Total Bilirubin | 0.7 (0.4 – 1.1) | 0.7 (0.4 – 1.1) | 0.7 (0.4 – 1.1) | 0.7 (0.5 – 1.1) | 0.8 (0.5 – 1.2) | 0.7 (0.5 – 1.1) | 0.6 (0.4 – 0.9) | 0.04 |
| Hematocrit | 30.7 (27.2 – 34.9) | 30.6 (27.0 – 34.9) | 30.8 (27.0 – 35.3) | 30.6 (27.1 – 35.3) | 30.2 (27.3 – 35.0) | 30.4 (27.0 – 34.7) | 30.1 (27.0 – 34.5) | 0.86 |
| Donor Risk Index | 1.85 (1.57 – 2.16) | 1.86 (1.61 – 2.16) | 1.85 (1.59 – 2.12) | 1.85 (1.60 – 2.11) | 1.83 (1.55 – 2.09) | 1.71 (1.53 – 2.00) | 1.83 (1.54 – 2.11) | 0.004 |
|  |  |  |  |  |  |  |  |  |
| Cold Ischemic Time (hours) | 7.0 ± 3.0  6.5 (5.0 – 8.3) | 7.0 ± 2.8  6.5 (5.2 – 8.4) | 7.2 ± 2.9  6.9 (5.3 – 8.6) | 7.2 ± 2.8  6.8 (5.3 – 8.5) | 7.3 ± 2.6  6.9 (5.5 – 9.0) | 7.2 ± 2.7  7.0 (5.2 – 8.6) | 7.1 ± 3.8  6.5 (5.2 – 8.4) | 0.004 |
| CIT Groups |  |  |  |  |  |  |  | 0.51 |
| <8 hours | 6,686 (67.9%) | 1,794 (68.1%) | 718 (65.3%) | 490 (65.6%) | 138 (62.7%) | 92 (62.2%) | 97 (69.3%) |  |
| 8 to 12 hours | 2,718 (27.6%) | 727 (27.6%) | 333 (30.3%) | 220 (29.5%) | 71 (32.3%) | 50 (33.8%) | 39 (27.9%) |  |
| ≥12 hours | 439 (4.5%) | 112 (4.3%) | 49 (4.5%) | 37 (5.0%) | 11 (5.0%) | 6 (4.1%) | 4 (2.9%) |  |
|  |  |  |  |  |  |  |  |  |
| Age | 55.6 ± 9.1  57 (51 – 62) | 55.7 ± 8.8  57 (51 – 62) | 56.3 ± 8.2  57 (52 – 62) | 55.5 ± 9.5  57 (51 – 62) | 56.4 ± 8.6  51 (46 – 57) | 54.2 ± 9.3  55 (50 – 60) | 54.9 ± 9.3  56 (51 – 60) | 0.12 |
| Age Groups |  |  |  |  |  |  |  | 0.26 |
| <35 years | 326 (3.3%) | 78 (2.9%) | 22 (2.0%) | 30 (3.9%) | 4 (1.8%) | 6 (4.0%) | 8 (5.6%) |  |
| 35 to 44 years | 670 (6.7%) | 172 (6.4%) | 66 (5.9%) | 53 (7.0%) | 13 (5.8%) | 13 (8.7%) | 6 (4.2%) |  |
| 45 to 54 years | 2,900 (29.0%) | 788 (29.5%) | 322 (28.7%) | 219 (28.7%) | 71 (31.8%) | 50 (33.3%) | 45 (31.7%) |  |
| 55 to 64 years | 4,628 (46.3%) | 1,269 (47.5%) | 547 (48.8%) | 340 (44.6%) | 94 (42.2%) | 66 (44.0%) | 64 (45.1%) |  |
| ≥65 years | 1,475 (14.8%) | 366 (13.7%) | 165 (14.7%) | 120 (15.8%) | 41 (18.4%) | 15 (10.0%) | 19 (13.4%) |  |
| Gender (female) | 3,012 (30.1%) | 705 (26.4%) | 301 (26.8%) | 182 (23.9%) | 54 (24.2%) | 42 (28.0%) | 43 (30.3%) | <0.001 |
| Ethnicity |  |  |  |  |  |  |  | 0.10 |
| White | 7,446 (74.5%) | 1,935 (72.4%) | 833 (74.2%) | 587 (77.0%) | 176 (78.9%) | 111 (74.0%) | 106 (74.7%) |  |
| Black | 897 (9.0%) | 255 (9.5%) | 94 (8.4%) | 52 (6.8%) | 10 (4.5%) | 17 (11.3%) | 18 (12.7%) |  |
| Hispanic | 1,118 (11.2%) | 309 (11.6%) | 126 (11.2%) | 81 (10.6%) | 24 (10.8%) | 17 (11.3%) | 10 (7.0%) |  |
| Asian | 390 (3.9%) | 136 (5.1%) | 54 (4.8%) | 35 (4.6%) | 10 (4.5%) | 4 (2.7%) | 4 (2.8%) |  |
| Other | 148 (1.5%) | 38 (1.4%) | 15 (1.3%) | 7 (0.9%) | 3 (1.4%) | 1 (0.7%) | 4 (2.8%) |  |
| Body Mass Index | 28.7 ± 5.6  28.1 (24.6 – 32.3) | 28.8 ± 5.6  28.2 (24.8 – 32.3) | 29.0 ± 5.7  28.5 (25.0 – 32.8) | 28.9 ± 6.0  27.9 (24.7 – 32.6) | 28.8 ± 5.7  27.7 (25.1 – 32.9) | 28.5 ± 5.6  28.1 (24.5 – 31.7) | 29.0 ± 5.8  28.9 (24.9 – 32.8) | 0.58 |
| BMI ≥30 kg/m^2^ | 3,799 (38.0%) | 1,024 (38.3%) | 459 (40.9%) | 278 (36.5%) | 76 (34.1%) | 54 (36.0%) | 61 (43.0%) | 0.25 |
| Blood Type |  |  |  |  |  |  |  | 0.005 |
| O | 4,605 (46.1%) | 1,192 (44.6%) | 503 (44.8%) | 381 (50.0%) | 99 (44.4%) | 73 (48.7%) | 70 (49.3%) |  |
| A | 3,637 (36.4%) | 979 (36.6%) | 440 (39.2%) | 276 (36.2%) | 92 (41.3%) | 53 (35.3%) | 50 (35.2%) |  |
| B | 1,265 (12.7%) | 396 (14.8%) | 137 (12.2%) | 81 (10.6%) | 29 (13.0%) | 17 (11.3%) | 19 (13.4%) |  |
| AB | 492 (4.9%) | 106 (4.0%) | 42 (3.7%) | 24 (3.2%) | 3 (1.4%) | 7 (4.7%) | 3 (2.1%) |  |
| MELD Score^a^ | 22.3 ± 9.5  22 (15 – 29) | 22.4 ± 9.7  22 (15 – 29) | 22.0 ± 9.4  21 (14 – 29) | 21.9 ± 9.2  21 (15 – 28) | 22.4 ± 9.7  21 (15 – 28) | 21.6 ± 8.7  21 (15 – 27) | 21.7 ± 8.5  20 (15 – 27) | 0.68 |
| MELD Groups |  |  |  |  |  |  |  | 0.06 |
| Low MELD  (Score <33) | 8.473 (84.9%) | 2,250 (84.3%) | 968 (86.3%) | 666 (87.6%) | 188 (84.3%) | 136 (90.7%) | 126 (88.7%) |  |
| Exceptions Given |  |  |  |  |  |  |  | 0.65 |
| No Exception | 6,097 (61.0%) | 1,611 (60.3%) | 692 (61.7%) | 490 (64.3%) | 139 (62.3%) | 101 (67.3%) | 88 (62.0%) |  |
| HCC Exception | 2,893 (28.9%) | 781 (29.2%) | 306 (27.3%) | 196 (25.7%) | 61 (27.4%) | 34 (22.7%) | 41 (28.9%) |  |
| Other Exception | 1,009 (10.1%) | 281 (10.5%) | 124 (11.1%) | 76 (10.0%) | 23 (10.3%) | 15 (10.0%) | 13 (9.2%) |  |
| Etiology of ESLD^b^ |  |  |  |  |  |  |  | 0.60 |
| Acute Liver Failure | 138 (1.4%) | 30 (1.1%) | 10 (0.9%) | 14 (1.8%) | 1 (0.5%) | 2 (1.3%) | 2 (1.4%) |  |
| CC/NASH | 1,206 (12.1%) | 330 (12.4%) | 154 (13.7%) | 109 (14.3%) | 31 (13.9%) | 15 (10.0%) | 21 (14.8%) |  |
| Cholestatic Disease | 704 (7.0%) | 200 (7.5%) | 62 (5.5%) | 61 (8.0%) | 18 (8.1%) | 11 (7.3%) | 10 (7.0%) |  |
| Cirrhosis (NOS) | 410 (4.1%) | 116 (4.3%) | 48 (4.3%) | 26 (3.4%) | 9 (4.0%) | 7 (4.7%) | 9 (6.3%) |  |
| Congenital/Metabolic | 305 (3.1%) | 56 (2.1%) | 27 (2.4%) | 15 (2.0%) | 2 (0.9%) | 5 (3.3%) | 3 (2.1%) |  |
| Alcohol | 1,262 (12.6%) | 357 (13.4%) | 150 (13.4%) | 104 (13.7%) | 32 (14.4%) | 25 (16.7%) | 18 (12.7%) |  |
| HBV | 144 (1.4%) | 43 (1.6%) | 20 (1.8%) | 14 (1.8%) | 3 (1.4%) | 2 (1.3%) | 2 (1.4%) |  |
| HCV | 2,795 (28.3%) | 735 (27.5%) | 301 (26.8%) | 190 (24.9%) | 51 (22.9%) | 42 (28.0%) | 38 (26.8%) |  |
| HCC | 2,833 (28.3%) | 759 (28.4%) | 321 (28.6%) | 217 (28.5%) | 73 (32.7%) | 39 (26.0%) | 38 (26.8%) |  |
| Other | 202 (2.0%) | 47 (1.8%) | 29 (2.6%) | 12 (1.6%) | 3 (1.4%) | 2 (1.3%) | 1 (0.7%) |  |
| Diabetes | 2,586 (26.2%) | 677 (25.6%) | 292 (26.2%) | 201 (26.5%) | 55 (24.8%) | 30 (20.1%) | 39 (28.1%) | 0.72 |
| Prior Malignancy | 1,910 (19.1%) | 525 (19.6%) | 225 (20.1%) | 136 (17.9%) | 45 (20.2%) | 19 (12.7%) | 34 (23.9%) | 0.23 |
| EBV-Positive | 6,283 (62.8%) | 1,727 (64.6%) | 703 (62.7%) | 506 (66.4%) | 141 (63.2%) | 91 (60.7%) | 83 (58.5%) | 0.23 |
| CMV-Positive | 6,032 (66.4%) | 1,607 (66.0%) | 651 (63.7%) | 463 (66.5%) | 121 (62.4%) | 79 (60.3%) | 81 (62.8%) | 0.34 |
| Prior Abdominal Surgery | 4,295 (43.7%) | 1,135 (43.2%) | 493 (44.7%) | 299 (39.9%) | 98 (44.6%) | 66 (44.9%) | 66 (47.1%) | 0.45 |
| Prior TIPS | 901 (9.2%) | 282 (10.7%) | 111 (10.0%) | 76 (10.1%) | 17 (7.7%) | 12 (8.2%) | 17 (12.2%) | 0.18 |
| PV Thrombosis | 1,005 (10.2%) | 281 (10.7%) | 109 (9.8%) | 75 (9.9%) | 23 (10.4%) | 15 (10.1%) | 15 (10.6%) | 0.99 |
| Encephalopathy | 6,047 (60.5%) | 1,592 (59.6%) | 673 (60.0%) | 453 (59.5%) | 133 (59.6%) | 93 (62.0%) | 86 (60.6%) | 0.98 |
| Ascites | 7,436 (74.4%) | 1,952 (73.0%) | 827 (73.7%) | 546 (71.7%) | 155 (69.5%) | 116 (77.3%) | 113 (79.6%) | 0.14 |
| Dialysis within 1 week of Transplant | 612 (6.1%) | 175 (6.6%) | 76 (6.8%) | 42 (5.5%) | 12 (5.4%) | 6 (4.0%) | 4 (2.8%) | 0.40 |
| Ventilator Support at Transplant | 264 (2.6%) | 65 (2.4%) | 23 (2.1%) | 12 (1.6%) | 7 (3.1%) | 1 (0.7%) | 3 (2.1%) | 0.32 |
|  |  |  |  |  |  |  |  |  |
| Region of Transplant |  |  |  |  |  |  |  | <0.001 |
| 1 | 471 (4.7%) | 134 (5.0%) | 65 (5.8%) | 77 (10.1%) | 19 (8.5%) | 13 (8.7%) | 9 (6.3%) |  |
| 2 | 1,323 (13.2%) | 387 (14.5%) | 151 (13.5%) | 69 (9.1%) | 33 (14.8%) | 19 (12.7%) | 7 (4.9%) |  |
| 3 | 1,294 (12.9%) | 280 (10.5%) | 154 (13.7%) | 108 (14.2%) | 28 (12.6%) | 18 (12.0%) | 16 (11.3%) |  |
| 4 | 882 (8.8%) | 177 (6.6%) | 72 (6.4%) | 32 (4.2%) | 13 (5.8%) | 14 (9.3%) | 7 (4.9%) |  |
| 5 | 872 (8.7%) | 333 (12.5%) | 145 (12.9%) | 98 (12.9%) | 31 (13.9%) | 10 (6.7%) | 9 (6.3%) |  |
| 6 | 388 (3.9%) | 105 (3.9%) | 35 (3.1%) | 6 (0.8%) | 1 (0.5%) | 2 (1.3%) | 0 (0.0%) |  |
| 7 | 1,008 (10.1%) | 252 (9.4%) | 102 (9.1%) | 48 (6.3%) | 18 (8.1%) | 6 (4.0%) | 6 (4.2%) |  |
| 8 | 938 (9.4%) | 216 (8.1%) | 88 (7.8%) | 67 (8.8%) | 14 (6.3%) | 16 (10.7%) | 5 (3.2%) |  |
| 9 | 704 (7.0%) | 285 (10.7%) | 111 (9.9%) | 111 (14.6%) | 27 (12.1%) | 14 (9.3%) | 13 (9.1%) |  |
| 10 | 650 (6.5%) | 200 (7.5%) | 68 (6.1%) | 45 (5.9%) | 22 (9.9%) | 13 (8.7%) | 7 (4.9%) |  |
| 11 | 1,469 (14.7%) | 304 (11.4%) | 131 (11.7%) | 101 (13.3%) | 17 (7.6%) | 25 (16.7%) | 63 (44.4%) |  |
