# Supplementary material for: Higher thresholds for the utilization of steatotic allografts in liver transplantation: Analysis from a U.S. national database
Source: PLoS One. 2020 Apr 2;15(4):e0230995. doi: 10.1371/journal.pone.0230995 (PMC7117730; doi:10.1371/journal.pone.0230995)
Supplement: S3 Table — Multivariable regression analyses for 30-Day (A), 90-Day (B), and 1-Year (C) graft loss. (DOCX) [file pone.0230995.s003.docx]

Table S3A. Logistic Regression Modeling for 30-Day Graft Loss Amongst All Organs with Biopsy

|  | Unadjusted | | | Adjusted^a^ | | |
| --- | --- | --- | --- | --- | --- | --- |
|  | OR | 95% CI | p-Value | OR | 95% CI | p-Value |
| Donor Variables |  |  |  |  |  |  |
| Biopsy Result- % MaS |  |  | <0.001 |  |  | <0.001 |
| 0 to 9% | Reference |  |  | Reference |  |  |
| 10 to 19% | 1.273 | 1.041 – 1.557 |  | 1.301 | 1.055 – 1.605 |  |
| 20 to 29% | 1.351 | 1.020 – 1.786 |  | 1.311 | 0.972 – 1.768 |  |
| 30 to 39% | 1.564 | 1.142 – 2.143 |  | 1.488 | 1.061 – 2.088 |  |
| 40 to 49% | 2.553 | 1.610 – 4.047 |  | 2.466 | 1.522 – 3.993 |  |
| 50 to 59% | 2.925 | 1.724 – 4.960 |  | 2.921 | 1.672 – 5.103 |  |
| ≥60% | 1.645 | 0.813 – 3.256 |  | 1.731 | 0.862 – 3.477 |  |
| Cause of Death |  |  | 0.004 |  |  | 0.005 |
| Anoxia | Reference |  |  | Reference |  |  |
| Trauma | 1.288 | 1.024 – 1.619 |  | 1.364 | 1.065 – 1.746 |  |
| CVA | 1.415 | 1.168 – 1.716 |  | 1.439 | 1.167 – 1.773 |  |
| Other | 1.450 | 0.888 – 2.365 |  | 1.558 | 0.931 – 2.606 |  |
| Cold Ischemic Time |  |  | <0.001 |  |  | <0.001 |
| <8 Hours | Reference |  |  | Reference |  |  |
| 8 to 12 Hours | 1.567 | 1.331 – 1.845 |  | 1.407 | 1.179 – 1.679 |  |
| ≥12 Hours | 2.465 | 1.857 – 3.271 |  | 2.402 | 1.757 – 3.283 |  |
| Recipient Variables |  |  |  |  |  |  |
| Body Mass Index ≥30 kg/m^2^ | 1.405 | 1.209 – 1.632 | <0.001 | 1.311 | 1.115 – 1.543 | 0.001 |
| Prior Abdominal Surgery | 1.521 | 1.308 – 1.769 | <0.001 | 1.462 | 1.242 – 1.719 | <0.001 |
| Portal Vein Thrombosis | 2.061 | 1.692 – 2.511 | <0.001 | 1.959 | 1.584 – 2.423 | <0.001 |
| Exception Points |  |  | <0.001 |  |  | 0.03 |
| No Exceptions | Reference |  |  | Reference |  |  |
| HCC Exception Points | 0.732 | 0.611 – 0.877 |  | 0.972 | 0.796 – 1.187 |  |
| Other Exception Points | 1.132 | 0.896 – 1.430 |  | 1.397 | 1.081 – 1.804 |  |
| On Vent at Transplant | 3.768 | 2.824 – 5.027 | <0.001 | 3.614 | 2.584 – 5.057 | <0.001 |
| On Dialysis at Transplant | 1.825 | 1.426 – 2.336 | <0.001 | 1.591 | 1.186 – 2.135 | 0.003 |
| Region of Transplant |  |  | <0.001 |  |  | <0.001 |
| 1 | Reference |  |  | Reference |  |  |
| 2 | 0.665 | 0.463 – 0.955 |  | 0.650 | 0.440 – 0.960 |  |
| 3 | 0.531 | 0.362 – 0.778 |  | 0.602 | 0.401 – 0.904 |  |
| 4 | 0.814 | 0.552 – 1.199 |  | 0.859 | 0.568 – 1.301 |  |
| 5 | 0.553 | 0.372 – 0.822 |  | 0.454 | 0.294 – 0.702 |  |
| 6 | 0.453 | 0.251 – 0.817 |  | 0.439 | 0.235 – 0.818 |  |
| 7 | 0.858 | 0.595 – 1.238 |  | 0.840 | 0.566 – 1.248 |  |
| 8 | 0.720 | 0.488 – 1.062 |  | 0.796 | 0.528 – 1.200 |  |
| 9 | 1.239 | 0.866 – 2.823 |  | 1.059 | 0.720 – 1.557 |  |
| 10 | 0.856 | 0.575 – 1.275 |  | 0.844 | 0.548 – 1.298 |  |
| 11 | 0.740 | 0.519 – 1.055 |  | 0.737 | 0.501 – 1.083 |  |
| ^a^- Adjusted scores based on multivariable models. All factors in multivariable models included in tables. | | | | | | |

Table S3B. Logistic Regression Modeling for 90-Day Graft Loss Amongst All Organs with Biopsy

|  | Unadjusted | | | Adjusted^a^ | | |
| --- | --- | --- | --- | --- | --- | --- |
|  | OR | 95% CI | p-Value | OR | 95% CI | p-Value |
| Donor Variables |  |  |  |  |  |  |
| Biopsy Result- % MaS |  |  | <0.001 |  |  | <0.001 |
| 0 to 9% | Reference |  |  | Reference |  |  |
| 10 to 19% | 1.144 | 0.968 – 1.353 |  | 1.147 | 0.964 – 1.368 |  |
| 20 to 29% | 1.277 | 1.013 – 1.608 |  | 1.231 | 0.963 – 1.573 |  |
| 30 to 39% | 1.360 | 1.040 – 1.778 |  | 1.317 | 0.990 – 1.751 |  |
| 40 to 49% | 2.150 | 1.435 – 3.223 |  | 2.108 | 1.383 – 3.213 |  |
| 50 to 59% | 2.155 | 1.323 – 3.511 |  | 2.257 | 1.350 – 3.771 |  |
| ≥60% | 1.361 | 0.749 – 2.472 |  | 1.396 | 0.758 – 2.571 |  |
| Cause of Death |  |  | <0.001 |  |  | <0.001 |
| Anoxia | Reference |  |  | Reference |  |  |
| Trauma | 1.199 | 0.989 – 1.453 |  | 1.281 | 1.041 – 1.577 |  |
| CVA | 1.518 | 1.296 – 1.778 |  | 1.485 | 1.249 – 1.766 |  |
| Other | 1.436 | 0.955 – 2.159 |  | 1.489 | 0.964 – 2.299 |  |
| Cold Ischemic Time |  |  | <0.001 |  |  | <0.001 |
| <8 Hours | Reference |  |  | Reference |  |  |
| 8 to 12 Hours | 1.479 | 1.293 – 1.691 |  | 1.348 | 1.166 – 1.560 |  |
| ≥12 Hours | 2.124 | 1.662 – 2.715 |  | 2.014 | 1.128 – 1.977 |  |
| CDC High Risk | 0.632 | 0.509 – 0.785 | <0.001 | 0.707 | 0.559 – 0.894 | 0.003 |
| Prior Myocardial Infarction | 1.306 | 1.034 – 1.651 | 0.03 | 1.325 | 1.029 – 1.707 | 0.03 |
| Recipient Variables |  |  |  |  |  |  |
| MELD Groups |  |  | <0.001 |  |  | 0.007 |
| Low MELD | Reference |  |  | Reference |  |  |
| High MELD | 1.608 | 1.383 – 1.871 |  | 1.344 | 1.089 – 1.659 |  |
| Body Mass Index ≥30 kg/m^2^ | 1.330 | 1.176 – 1.505 | <0.001 | 1.251 | 1.094 – 1.429 | 0.001 |
| Prior Abdominal Surgery | 1.379 | 1.219 – 1.561 | <0.001 | 1.350 | 1.182 – 1.541 | <0.001 |
| Portal Vein Thrombosis | 1.869 | 1.579 – 2.213 | <0.001 | 1.820 | 1.518 – 2.181 | <0.001 |
| Exception Points |  |  | <0.001 |  |  | 0.03 |
| No Exceptions | Reference |  |  | Reference |  |  |
| HCC Exception Points | 0.710 | 0.612 – 0.824 |  | 0.967 | 0.817 – 1.146 |  |
| Other Exception Points | 1.051 | 0.863 – 1.279 |  | 1.326 | 1.065 – 1.651 |  |
| On Vent at Transplant | 3.641 | 2.835 – 4.676 | <0.001 | 3.125 | 2.337 – 4.179 | <0.001 |
| On Dialysis at Transplant | 1.950 | 1.594 – 2.384 | <0.001 | 1.464 | 1.120 – 1.915 | 0.006 |
| Region of Transplant |  |  | <0.001 |  |  | 0.001 |
| 1 | Reference |  |  | Reference |  |  |
| 2 | 0.739 | 0.546 – 1.000 |  | 0.728 | 0.524 – 1.011 |  |
| 3 | 0.698 | 0.512 – 0.952 |  | 0.790 | 0.566 – 1.102 |  |
| 4 | 0.763 | 0.547 – 1.065 |  | 0.823 | 0.575 – 1.176 |  |
| 5 | 0.548 | 0.392 – 0.768 |  | 0.467 | 0.323 – 0.676 |  |
| 6 | 0.507 | 0.314 – 0.817 |  | 0.512 | 0.310 – 0.845 |  |
| 7 | 0.886 | 0.650 – 1.208 |  | 0.860 | 0.614 – 1.204 |  |
| 8 | 0.703 | 0.505 – 0.978 |  | 0.779 | 0.548 – 1.107 |  |
| 9 | 1.367 | 1.011 – 1.848 |  | 1.232 | 0.889 – 1.706 |  |
| 10 | 0.927 | 0.664 – 1.294 |  | 0.949 | 0.661 – 1.364 |  |
| 11 | 0.826 | 0.614 – 1.111 |  | 0.851 | 0.616 – 1.177 |  |

Table S3C. Logistic Regression Modeling for 1-Year Graft Loss Amongst All Organs with Biopsy

|  | Unadjusted | | | Adjusted^a^ | | |
| --- | --- | --- | --- | --- | --- | --- |
|  | OR | 95% CI | p-Value | OR | 95% CI | p-Value |
| Donor Variables |  |  |  |  |  |  |
| Age Groups |  |  | <0.001 |  |  | <0.001 |
| <35 Years | Reference |  |  | Reference |  |  |
| 35 to 44 Years | 1.352 | 1.130 – 1.619 |  | 1.304 | 1.073 – 1.585 |  |
| 45 to 54 Years | 1.469 | 1.253 – 1.722 |  | 1.356 | 1.135 – 1.621 |  |
| 55 to 64 Years | 1.752 | 1.496 – 2.053 |  | 1.596 | 1.332 – 1.912 |  |
| ≥65 Years | 1.739 | 1.469 – 2.059 |  | 1.554 | 1.276 – 1.892 |  |
| Biopsy Result- % MaS |  |  | 0.095 |  |  | 0.03 |
| 0 to 9% | Reference |  |  | Reference |  |  |
| 10 to 19% | 0.987 | 0.867 – 1.123 |  | 0.964 | 0.841 – 1.104 |  |
| 20 to 29% | 1.108 | 0.924 – 1.329 |  | 1.106 | 0.913 – 1.339 |  |
| 30 to 39% | 1.088 | 0.876 – 1.352 |  | 1.070 | 0.850 – 1.346 |  |
| 40 to 49% | 1.415 | 0.982 – 2.040 |  | 1.458 | 0.997 – 2.131 |  |
| 50 to 59% | 1.710 | 1.131 – 2.587 |  | 1.964 | 1.272 – 3.032 |  |
| ≥60% | 1.179 | 0.730 – 1.906 |  | 1.335 | 0.815 – 2.187 |  |
| Cause of Death |  |  | <0.001 |  |  | <0.001 |
| Anoxia | Reference |  |  | Reference |  |  |
| Trauma | 1.053 | 0.913 – 1.215 |  | 1.171 | 1.002 – 1.368 |  |
| CVA | 1.367 | 1.217 – 1.536 |  | 1.324 | 1.160 – 1.510 |  |
| Other | 1.239 | 0.907 – 1.692 |  | 1.331 | 0.948 – 1.868 |  |
| EBV Positive | 0.870 | 0.766 – 0.989 | 0.03 | 0.830 | 0.719 – 0.958 | 0.01 |
| Cold Ischemic Time |  |  | <0.001 |  |  | <0.001 |
| <8 Hours | Reference |  |  | Reference |  |  |
| 8 to 12 Hours | 1.134 | 1.021 – 1.258 |  | 1.087 | 0.970 – 1.218 |  |
| ≥12 Hours | 1.708 | 1.403 – 2.079 |  | 1.637 | 1.311 – 2.045 |  |
| Recipient Variables |  |  |  |  |  |  |
| Age Groups |  |  | <0.001 |  |  | <0.001 |
| <35 Years | Reference |  |  | Reference |  |  |
| 35 to 44 Years | 1.284 | 1.103 – 1.494 |  | 0.947 | 0.647 – 1.387 |  |
| 45 to 54 Years | 0.975 | 0.839 – 1.133 |  | 1.106 | 0.792 – 1.546 |  |
| 55 to 64 Years | 1.205 | 0.901 – 1.614 |  | 1.192 | 0.857 – 1.657 |  |
| ≥65 Years | 1.536 | 1.133 – 2.084 |  | 1.542 | 1.094 – 2.175 |  |
| Ethnicity |  |  | 0.006 |  |  | 0.02 |
| White | Reference |  |  | Reference |  |  |
| Black | 1.284 | 1.103 – 1.494 |  | 1.345 | 1.136 – 1.593 |  |
| Hispanic | 0.975 | 0.839 – 1.133 |  | 0.971 | 0.820 – 1.150 |  |
| Asian | 0.861 | 0.672 – 1.104 |  | 0.978 | 0.738 – 1.296 |  |
| Other | 0.746 | 0.479 – 1.164 |  | 0.920 | 0.582 – 1.455 |  |
| MELD Groups |  |  | <0.001 |  |  | <0.001 |
| Low MELD | Reference |  |  | Reference |  |  |
| High MELD | 1.549 | 1.377 – 1.743 |  | 1.345 | 1.148 – 1.576 |  |
| Prior Abdominal Surgery | 1.303 | 1.187 – 1.430 | <0.001 | 1.273 | 1.150 – 1.409 | <0.001 |
| Portal Vein Thrombosis | 1.580 | 1.377 – 1.814 | <0.001 | 1.583 | 1.365 – 1.836 | <0.001 |
| On Vent at Transplant | 2.877 | 2.388 – 3.466 | <0.001 | 2.786 | 2.171 – 3.576 | <0.001 |
| On Dialysis at Transplant | 1.864 | 1.587 – 2.189 | <0.001 | 1.444 | 1.168 – 1.786 | <0.001 |
| Encephalopathy | 1.203 | 1.092 – 1.324 | <0.001 | 1.160 | 1.038 – 1.295 | 0.008 |
| Etiology of ESLD |  |  | 0.02 |  |  | 0.006 |
| Acute | Reference |  |  | Reference |  |  |
| CC/NASH | 1.112 | 0.727 – 1.701 |  | 0.955 | 0.604 – 1.511 |  |
| Cholestatic | 0.821 | 0.525 – 1.283 |  | 0.815 | 0.505 – 1.317 |  |
| Cirrhosis (NOS) | 1.073 | 0.676 – 1.704 |  | 0.890 | 0.539 – 1.470 |  |
| Congenital/Metabolic | 0.966 | 0.588 – 1.588 |  | 0.923 | 0.540 – 1.578 |  |
| Alcohol | 0.865 | 0.564 – 1.327 |  | 0.807 | 0.509 – 1.279 |  |
| HBV | 0.708 | 0.392 – 1.279 |  | 0.705 | 0.369 – 1.346 |  |
| HCV | 1.064 | 0.703 – 1.611 |  | 1.116 | 0.714 – 1.743 |  |
| HCC | 0.982 | 0.648 – 1.487 |  | 1.073 | 0.685 – 1.681 |  |
| Other | 1.102 | 0.660 – 1.839 |  | 1.230 | 0.704 – 2.148 |  |
| Region of Transplant |  |  | <0.001 |  |  | <0.001 |
| 1 | Reference |  |  | Reference |  |  |
| 2 | 0.914 | 0.728 – 1.149 |  | 0.892 | 0.696 – 1.144 |  |
| 3 | 0.831 | 0.657 – 1.050 |  | 0.887 | 0.687 – 1.144 |  |
| 4 | 0.717 | 0.553 – 0.929 |  | 0.743 | 0.561 – 0.984 |  |
| 5 | 0.687 | 0.536 – 0.881 |  | 0.603 | 0.456 – 0.796 |  |
| 6 | 0.577 | 0.405 – 0.823 |  | 0.608 | 0.415 – 0.890 |  |
| 7 | 0.849 | 0.667 – 1.080 |  | 0.873 | 0.671 – 1.135 |  |
| 8 | 0.709 | 0.550 – 0.914 |  | 0.807 | 0.615 – 1.060 |  |
| 9 | 1.176 | 0.926 – 1.494 |  | 1.114 | 0.859 – 1.444 |  |
| 10 | 0.861 | 0.663 – 1.118 |  | 0.897 | 0.673 – 1.194 |  |
| 11 | 0.763 | 0.605 – 0.961 |  | 0.787 | 0.610 – 1.015 |  |
