# Supplementary material for: Higher thresholds for the utilization of steatotic allografts in liver transplantation: Analysis from a U.S. national database
Source: PLoS One. 2020 Apr 2;15(4):e0230995. doi: 10.1371/journal.pone.0230995 (PMC7117730; doi:10.1371/journal.pone.0230995)
Supplement: S4 Table — Multivariable regression analyses for 30-Day (A), 90-Day (B), and 1-Year (C) graft loss amongst recipients with MELD Score <33. Factors known to influence allograft outcomes forced into models (ie. donor age, recipient age, etiology of ESLD). (DOCX) [file pone.0230995.s004.docx]

Table S4A. Logistic Regression Modeling for 30-Day Graft Loss in Lower MELD Recipients

|  | Unadjusted | | | Adjusted^a^ | | |
| --- | --- | --- | --- | --- | --- | --- |
|  | OR | 95% CI | p-Value | OR | 95% CI | p-Value |
| Donor Variables |  |  |  |  |  |  |
| Age Groups |  |  | 0.0625 |  |  | 0.5373 |
| <35 Years | Reference |  |  | Reference |  |  |
| 35 to 44 Years | 1.318 | 0.961 – 1.807 |  | 1.225 | 0.875 – 1.715 |  |
| 45 to 54 Years | 1.414 | 1.069 – 1.868 |  | 1.147 | 0.840 – 1.566 |  |
| 55 to 64 Years | 1.203 | 0.900 – 1.609 |  | 0.978 | 0.705 – 1.356 |  |
| ≥65 Years | 1.480 | 1.098 – 1.994 |  | 1.066 | 0.752 – 1.513 |  |
| Biopsy Result- % MaS |  |  | 0.0002 |  |  | 0.0021 |
| 0 to 9% | Reference |  |  | Reference |  |  |
| 10 to 19% | 1.323 | 1.058 – 1.655 |  | 1.368 | 1.085 – 1.725 |  |
| 20 to 29% | 1.491 | 1.103 – 2.016 |  | 1.373 | 0.993 – 1.898 |  |
| 30 to 39% | 1.458 | 1.018 – 2.087 |  | 1.280 | 0.869 – 1.885 |  |
| 40 to 49% | 1.961 | 1.103 – 3.484 |  | 1.807 | 0.984 – 3.319 |  |
| 50 to 59% | 3.036 | 1.726 – 5.341 |  | 2.962 | 1.627 – 5.391 |  |
| ≥60% | 1.779 | 0.862 – 3.673 |  | 1.715 | 0.815 – 3.607 |  |
| Cause of Death |  |  | 0.0116 |  |  | 0.0234 |
| Anoxia | Reference |  |  | Reference |  |  |
| Trauma | 1.312 | 1.014 – 1.698 |  | 1.376 | 1.041 – 1.818 |  |
| CVA | 1.426 | 1.147 – 1.772 |  | 1.424 | 1.115 – 1.820 |  |
| Other | 1.478 | 0.852 – 2.564 |  | 1.579 | 0.884 – 2.820 |  |
| CDC High Risk Donor | 0.604 | 0.445 – 0.820 | 0.0012 | 0.722 | 0.520 – 1.002 |  |
|  |  |  |  |  |  |  |
| Cold Ischemic Time |  |  | <0.0001 |  |  | <0.0001 |
| <8 Hours | Reference |  |  | Reference |  |  |
| 8 to 12 Hours | 1.567 | 1.303 – 1.884 |  | 1.405 | 1.152 – 1.713 |  |
| ≥12 Hours | 2.488 | 1.824 – 3.395 |  | 2.286 | 1.619 – 3.226 |  |
| Recipient Variables |  |  |  |  |  |  |
| Age Groups |  |  | 0.5512 |  |  | 0.6958 |
| <35 Years | Reference |  |  | Reference |  |  |
| 35 to 44 Years | 0.830 | 0.470 – 1.467 |  | 0.930 | 0.502 – 1.721 |  |
| 45 to 54 Years | 0.773 | 0.474 – 1.260 |  | 0.806 | 0.468 – 1.389 |  |
| 55 to 64 Years | 0.781 | 0.484 – 1.259 |  | 0.834 | 0.489 – 1.423 |  |
| ≥65 Years | 0.925 | 0.559 – 1.530 |  | 0.966 | 0.552 – 1.5 |  |
| Body Mass Index >30 kg/m^2^ | 1.380 | 1.166 – 1.634 | 0.0002 | 1.323 | 1.099 – 1.594 | 0.0033 |
| Prior Abdominal Surgery | 1.527 | 1.288 – 1.810 | <0.0001 |  |  |  |
| PV Thrombosis | 2.015 | 1.612 – 2.517 | <0.0001 |  |  |  |
| Etiology of ESLD |  |  | 0.0360 |  |  | 0.6075 |
| Acute | Reference |  |  | Reference |  |  |
| CC/NASH | 1.177 | 0.536 – 2.585 |  | 0.749 | 0.333 – 1.685 |  |
| Cholestatic | 0.963 | 0.426 – 2.180 |  | 0.791 | 0.343 – 1.823 |  |
| Cirrhosis (NOS) | 0.852 | 0.356 – 2.037 |  | 0.618 | 0.251 – 1.525 |  |
| Congenital/Metabolic | 0.779 | 0.304 – 1.994 |  | 0.475 | 0.174 – 1.296 |  |
| Alcohol | 0.861 | 0.388 – 1.910 |  | 0.651 | 0.287 – 1.473 |  |
| HBV | 0.836 | 0.297 – 2.369 |  | 0.678 | 0.228 – 2.013 |  |
| HCV | 0.706 | 0.324 – 1.539 |  | 0.590 | 0.266 – 1.312 |  |
| HCC | 0.739 | 0.340 – 1.605 |  | 0.598 | 0.269 – 1.326 |  |
| Other | 1.071 | 0.431 – 2.666 |  | 0.823 | 0.319 – 2.123 |  |
| Region of Transplant |  |  | <0.0001 |  |  | <0.0001 |
| 1 | Reference |  |  | Reference |  |  |
| 2 | 0.630 | 0.420 – 0.943 |  | 0.654 | 0.423 – 1.011 |  |
| 3 | 0.464 | 0.303 – 0.710 |  | 0.531 | 0.339 – 0.833 |  |
| 4 | 0.773 | 0.504 – 1.185 |  | 0.825 | 0.521 – 1.306 |  |
| 5 | 0.445 | 0.277 – 0.715 |  | 0.386 | 0.228 – 0.653 |  |
| 6 | 0.359 | 0.178 – 0.725 |  | 0.370 | 0.180 – 0.762 |  |
| 7 | 0.834 | 0.554 – 1.256 |  | 0.866 | 0.557 – 1.345 |  |
| 8 | 0.668 | 0.434 – 1.030 |  | 0.722 | 0.457 – 1.141 |  |
| 9 | 1.278 | 0.865 – 1.890 |  | 1.096 | 0.718 – 1.673 |  |
| 10 | 0.831 | 0.539 – 1.281 |  | 0.772 | 0.480 – 1.240 |  |
| 11 | 0.737 | 0.502 – 1.083 |  | 0.720 | 0.472 – 1.097 |  |

Table S4B. Logistic Regression Modeling for 90-Day Graft Loss in Lower MELD Recipients

|  | Unadjusted | | | Adjusted^a^ | | |
| --- | --- | --- | --- | --- | --- | --- |
|  | OR | 95% CI | p-Value | OR | 95% CI | p-Value |
| Donor Variables |  |  |  |  |  |  |
| Age Groups |  |  | 0.0014 |  |  | 0.5487 |
| <35 Years | Reference |  |  | Reference |  |  |
| 35 to 44 Years | 1.339 | 1.027 – 1.746 |  | 1.259 | 0.950 – 1.668 |  |
| 45 to 54 Years | 1.398 | 1.105 – 1.770 |  | 1.142 | 0.879 – 1.483 |  |
| 55 to 64 Years | 1.410 | 1.110 – 1.791 |  | 1.106 | 0.845 – 1.449 |  |
| ≥65 Years | 1.675 | 1.309 – 2.143 |  | 1.199 | 0.899 – 1.598 |  |
| Biopsy Result- % MaS |  |  | 0.0094 |  |  | 0.0358 |
| 0 to 9% | Reference |  |  | Reference |  |  |
| 10 to 19% | 1.149 | 0.952 – 1.387 |  | 1.175 | 0.967 – 1.427 |  |
| 20 to 29% | 1.287 | 0.995 – 1.663 |  | 1.178 | 0.895 – 1.550 |  |
| 30 to 39% | 1.296 | 0.960 – 1.757 |  | 1.187 | 0.862 – 1.636 |  |
| 40 to 49% | 1.601 | 0.965 – 2.658 |  | 1.537 | 0.905 – 2.612 |  |
| 50 to 59% | 2.294 | 1.368 – 3.844 |  | 2.350 | 1.366 – 4.042 |  |
| ≥60% | 1.523 | 0.815 – 2.845 |  | 1.494 | 0.788 – 2.831 |  |
| Cause of Death |  |  | 0.0007 |  |  | 0.0151 |
| Anoxia | Reference |  |  | Reference |  |  |
| Trauma | 1.224 | 0.989 – 1.515 |  | 1.290 | 1.026 – 1.622 |  |
| CVA | 1.430 | 1.198 – 1.708 |  | 1.345 | 1.102 – 1.641 |  |
| Other | 1.480 | 0.943 – 2.321 |  | 1.595 | 0.997 – 2.553 |  |
| CDC High Risk Donor | 0.573 | 0.445 – 0.740 | <0.0001 | 0.655 | 0.496 – 0.865 | 0.0018 |
|  |  |  |  |  |  |  |
| Cold Ischemic Time |  |  | <0.0001 |  |  | <0.0001 |
| <8 Hours | Reference |  |  | Reference |  |  |
| 8 to 12 Hours | 1.432 | 1.230 – 1.667 |  | 1.294 | 1.099 – 1.525 |  |
| ≥12 Hours | 2.106 | 1.609 – 2.757 |  | 1.850 | 1.369 – 2.501 |  |
| Recipient Variables |  |  |  |  |  |  |
| Age Groups |  |  | 0.5392 |  |  | 0.8015 |
| <35 Years | Reference |  |  | Reference |  |  |
| 35 to 44 Years | 0.932 | 0.559 – 1.554 |  | 0.927 | 0.535 – 1.606 |  |
| 45 to 54 Years | 0.967 | 0.621 – 1.504 |  | 0.957 | 0.590 – 1.551 |  |
| 55 to 64 Years | 1.016 | 0.658 – 1.567 |  | 1.020 | 0.634 – 1.642 |  |
| ≥65 Years | 1.156 | 0.734 – 1.820 |  | 1.101 | 0.670 – 1.809 |  |
| Body Mass Index >30 kg/m^2^ | 1.320 | 1.148 – 1.517 | <0.0001 | 1.244 | 1.068 – 1.450 | 0.0052 |
| Prior Abdominal Surgery | 1.374 | 1.196 – 1.580 | <0.0001 | 1.340 | 1.153 – 1.557 | 0.0001 |
| PV Thrombosis | 1.754 | 1.446 – 2.128 | <0.0001 | 1.684 | 1.373 – 2.065 | <0.0001 |
| Etiology of ESLD |  |  | 0.0002 |  |  | 0.2213 |
| Acute | Reference |  |  | Reference |  |  |
| CC/NASH | 1.176 | 0.622 – 2.225 |  | 0.780 | 0.404 – 1.505 |  |
| Cholestatic | 0.781 | 0.399 – 1.526 |  | 0.640 | 0.322 – 1.272 |  |
| Cirrhosis (NOS) | 0.861 | 0.426 – 1.742 |  | 0.617 | 0.296 – 1.285 |  |
| Congenital/Metabolic | 0.878 | 0.416 – 1.853 |  | 0.564 | 0.256 – 1.244 |  |
| Alcohol | 0.867 | 0.455 – 1.652 |  | 0.685 | 0.353 – 1.326 |  |
| HBV | 0.589 | 0.237 – 1.462 |  | 0.488 | 0.188 – 1.263 |  |
| HCV | 0.674 | 0.358 – 1.267 |  | 0.569 | 0.298 – 1.088 |  |
| HCC | 0.702 | 0.374 – 1.317 |  | 0.583 | 0.306 – 1.113 |  |
| Other | 1.032 | 0.491 – 2.170 |  | 0.833 | 0.384 – 1.805 |  |
| Region of Transplant |  |  | <0.0001 |  |  | <0.0001 |
| 1 | Reference |  |  | Reference |  |  |
| 2 | 0.711 | 0.506 – 1.000 |  | 0.763 | 0.527 – 1.104 |  |
| 3 | 0.668 | 0.474 – 0.942 |  | 0.759 | 0.525 – 1.098 |  |
| 4 | 0.714 | 0.492 – 1.037 |  | 0.803 | 0.537 – 1.201 |  |
| 5 | 0.472 | 0.316 – 0.705 |  | 0.473 | 0.305 – 0.735 |  |
| 6 | 0.467 | 0.271 – 0.806 |  | 0.524 | 0.298 – 0.921 |  |
| 7 | 0.875 | 0.617 – 1.242 |  | 0.939 | 0.642 – 1.371 |  |
| 8 | 0.679 | 0.469 – 0.983 |  | 0.764 | 0.515 – 1.133 |  |
| 9 | 1.386 | 0.992 – 1.937 |  | 1.307 | 0.909 – 1.879 |  |
| 10 | 0.917 | 0.636 – 1.323 |  | 0.900 | 0.602 – 1.346 |  |
| 11 | 0.842 | 0.608 – 1.166 |  | 0.867 | 0.606 – 1.240 |  |

Table S4C. Logistic Regression Modeling for 1-Year Graft Loss in Lower MELD Recipients

|  | Unadjusted | | | Adjusted^a^ | | |
| --- | --- | --- | --- | --- | --- | --- |
|  | OR | 95% CI | p-Value | OR | 95% CI | p-Value |
| Donor Variables |  |  |  |  |  |  |
| Age Groups |  |  | <0.0001 |  |  | 0.0180 |
| <35 Years | Reference |  |  | Reference |  |  |
| 35 to 44 Years | 1.329 | 1.087 – 1.626 |  | 1.313 | 1.056 – 1.631 |  |
| 45 to 54 Years | 1.464 | 1.226 – 1.747 |  | 1.297 | 1.063 – 1.583 |  |
| 55 to 64 Years | 1.612 | 1.349 – 1.926 |  | 1.387 | 1.131 – 1.702 |  |
| ≥65 Years | 1.742 | 1.446 – 2.097 |  | 1.414 | 1.135 – 1.762 |  |
| Biopsy Result- % MaS |  |  | 0.2354 |  |  | 0.1443 |
| 0 to 9% | Reference |  |  | Reference |  |  |
| 10 to 19% | 0.960 | 0.829 – 1.111 |  | 0.950 | 0.817 – 1.105 |  |
| 20 to 29% | 1.062 | 0.866 – 1.302 |  | 1.032 | 0.833 – 1.278 |  |
| 30 to 39% | 1.094 | 0.863 – 1.389 |  | 1.037 | 0.807 – 1.334 |  |
| 40 to 49% | 1.141 | 0.737 – 1.765 |  | 1.150 | 0.732 – 1.808 |  |
| 50 to 59% | 1.718 | 1.103 – 2.675 |  | 1.871 | 1.178 – 2.970 |  |
| ≥60% | 1.359 | 0.828 – 2.232 |  | 1.501 | 0.903 – 2.494 |  |
| Cause of Death |  |  | <0.0001 |  |  | 0.0014 |
| Anoxia | Reference |  |  | Reference |  |  |
| Trauma | 1.094 | 0.933 – 1.282 |  | 1.197 | 1.007 – 1.422 |  |
| CVA | 1.363 | 1.197 – 1.553 |  | 1.337 | 1.154 – 1.550 |  |
| Other | 1.269 | 0.897 – 1.795 |  | 1.336 | 0.918 – 1.943 |  |
| CDC High Risk Donor | 0.713 | 0.599 – 0.849 | <0.0001 | 0.755 | 0.621 – 0.918 | 0.0039 |
| EBV-Positive | 0.823 | 0.717 – 0.944 | 0.0061 | 0.828 | 0.710 – 0.966 |  |
|  |  |  |  |  |  |  |
| Cold Ischemic Time |  |  | <0.0001 |  |  | 0.0013 |
| <8 Hours | Reference |  |  | Reference |  |  |
| 8 to 12 Hours | 1.096 | 0.975 – 1.232 |  | 1.029 | 0.907 – 1.169 |  |
| ≥12 Hours | 1.758 | 1.423 – 2.171 |  | 1.580 | 1.246 – 2.005 |  |
| Recipient Variables |  |  |  |  |  |  |
| Age Groups |  |  | 0.0012 |  |  | 0.0139 |
| <35 Years | Reference |  |  | Reference |  |  |
| 35 to 44 Years | 1.017 | 0.673 – 1.535 |  | 1.007 | 0.641 – 1.582 |  |
| 45 to 54 Years | 1.169 | 0.816 – 1.673 |  | 1.041 | 0.696 – 1.555 |  |
| 55 to 64 Years | 1.295 | 0.910 – 1.843 |  | 1.164 | 0.783 – 1.730 |  |
| ≥65 Years | 1.542 | 1.069 – 2.225 |  | 1.404 | 0.931 – 2.116 |  |
| Ethnicity |  |  | 0.0176 |  |  | 0.0193 |
| White | Reference |  |  | Reference |  |  |
| Black | 1.298 | 1.095 – 1.539 |  | 1.388 | 1.150 – 1.674 |  |
| Hispanic | 1.058 | 0.895 – 1.250 |  | 1.079 | 0.894 – 1.303 |  |
| Asian | 0.903 | 0.692 – 1.180 |  | 1.017 | 0.753 – 1.374 |  |
| Other | 0.714 | 0.432 – 1.180 |  | 0.886 | 0.531 – 1.480 |  |
| Prior Abdominal Surgery | 1.295 | 1.167 – 1.437 | <0.0001 | 1.287 | 1.150 – 1.441 | <0.0001 |
| PV Thrombosis | 1.500 | 1.285 – 1.752 | <0.0001 | 1.523 | 1.293 – 1.795 | <0.0001 |
| Encephalopathy | 1.159 | 1.043 – 1.287 | 0.0058 | 1.212 | 1.076 – 1.366 | 0.0015 |
| Etiology of ESLD |  |  | 0.0098 |  |  | 0.0027 |
| Acute | Reference |  |  | Reference |  |  |
| CC/NASH | 1.101 | 0.649 – 1.868 |  | 0.842 | 0.481 – 1.474 |  |
| Cholestatic | 0.773 | 0.445 – 1.344 |  | 0.676 | 0.378 – 1.211 |  |
| Cirrhosis (NOS) | 0.969 | 0.545 – 1.721 |  | 0.716 | 0.387 – 1.324 |  |
| Congenital/Metabolic | 0.904 | 0.489 – 1.670 |  | 0.730 | 0.380 – 1.402 |  |
| Alcohol | 0.862 | 0.506 – 1.469 |  | 0.716 | 0.408 – 1.258 |  |
| HBV | 0.443 | 0.200 – 0.981 |  | 0.362 | 0.152 – 0.863 |  |
| HCV | 1.063 | 0.633 – 1.784 |  | 0.973 | 0.563 – 1.682 |  |
| HCC | 0.981 | 0.585 – 1.646 |  | 0.884 | 0.512 – 1.529 |  |
| Other | 1.101 | 0.600 – 2.019 |  | 1.064 | 0.559 – 2.027 |  |
| Region of Transplant |  |  | <0.0001 |  |  | <0.0001 |
| 1 | Reference |  |  | Reference |  |  |
| 2 | 0.927 | 0.718 – 1.197 |  | 0.945 | 0.717 – 1.246 |  |
| 3 | 0.828 | 0.640 – 1.072 |  | 0.841 | 0.636 – 1.112 |  |
| 4 | 0.705 | 0.529 – 0.939 |  | 0.707 | 0.516 – 0.965 |  |
| 5 | 0.647 | 0.485 – 0.863 |  | 0.586 | 0.425 – 0.809 |  |
| 6 | 0.570 | 0.385 – 0.844 |  | 0.569 | 0.374 – 0.867 |  |
| 7 | 0.813 | 0.619 – 1.069 |  | 0.888 | 0.661 – 1.193 |  |
| 8 | 0.685 | 0.515 – 0.910 |  | 0.749 | 0.553 – 1.014 |  |
| 9 | 1.223 | 0.939 – 1.593 |  | 1.164 | 0.874 – 1.550 |  |
| 10 | 0.893 | 0.672 – 1.188 |  | 0.895 | 0.655 – 1.222 |  |
| 11 | 0.782 | 0.607 – 1.008 |  | 0.752 | 0.569 – 0.994 |  |
