# Supplementary material for: Higher thresholds for the utilization of steatotic allografts in liver transplantation: Analysis from a U.S. national database
Source: PLoS One. 2020 Apr 2;15(4):e0230995. doi: 10.1371/journal.pone.0230995 (PMC7117730; doi:10.1371/journal.pone.0230995)
Supplement: S5 Table — Multivariable regression analyses for 30-Day (A), 90-Day (B), and 1-Year (C) graft loss amongst recipients with MELD Scores 33 and higher. Factors known to influence allograft outcomes forced into models (ie. donor age, recipient age, etiology of ESLD). (DOCX) [file pone.0230995.s005.docx]

Table S5A. Logistic Regression Modeling for 30-Day Graft Loss in Higher MELD Recipients

|  | Unadjusted | | | Adjusted^a^ | | |
| --- | --- | --- | --- | --- | --- | --- |
|  | OR | 95% CI | p-Value | OR | 95% CI | p-Value |
| Donor Variables |  |  |  |  |  |  |
| Age Groups |  |  | 0.06 |  |  | 0.15 |
| <35 Years | Reference |  |  | Reference |  |  |
| 35 to 44 Years | 1.892 | 1.004 – 3.564 |  | 1.580 | 0.785 – 3.181 |  |
| 45 to 54 Years | 1.830 | 1.018 – 3.290 |  | 1.868 | 0.996 – 3.502 |  |
| 55 to 64 Years | 2.319 | 1.297 – 4.146 |  | 2.178 | 1.159 – 4.092 |  |
| ≥65 Years | 1.878 | 0.950 – 3.715 |  | 1.580 | 0.734 – 3.400 |  |
| Biopsy Result- % MaS |  |  | 0.007 |  |  | 0.003 |
| 0 to 9% | Reference |  |  | Reference |  |  |
| 10 to 19% | 1.077 | 0.680 – 1.707 |  | 0.991 | 0.604 – 1.627 |  |
| 20 to 29% | 0.808 | 0.367 – 1.780 |  | 0.847 | 0.376 – 1.909 |  |
| 30 to 39% | 2.250 | 1.156 – 4.380 |  | 2.365 | 1.163 – 4.810 |  |
| 40 to 49% | 5.223 | 2.295 – 11.888 |  | 6.878 | 2.631 – 17.984 |  |
| 50 to 59% | 2.829 | 0.623 – 12.847 |  | 2.552 | 0.500 – 13.034 |  |
| ≥60% | 1.132 | 0.148 – 8.671 |  | 1.383 | 0.168 – 11.394 |  |
|  |  |  |  |  |  |  |
| Cold Ischemic Time |  |  | 0.003 |  |  | 0.01 |
| <8 Hours | Reference |  |  | Reference |  |  |
| 8 to 12 Hours | 1.602 | 1.125 – 2.283 |  | 1.469 | 0.983 – 2.195 |  |
| ≥12 Hours | 2.863 | 1.421 – 5.768 |  | 3.204 | 1.450 – 7.082 |  |
| Recipient Variables |  |  |  |  |  |  |
| Age Groups |  |  | 0.40 |  |  | 0.65 |
| <35 Years | Reference |  |  | Reference |  |  |
| 35 to 44 Years | 1.277 | 0.478 – 3.406 |  | 1.090 | 0.381 – 3.115 |  |
| 45 to 54 Years | 1.248 | 0.522 – 2.981 |  | 1.180 | 0.466 – 2.989 |  |
| 55 to 64 Years | 1.331 | 0.564 – 3.143 |  | 1.249 | 0.501 – 3.113 |  |
| ≥65 Years | 2.033 | 0.806 – 5.127 |  | 1.794 | 0.664 – 4.848 |  |
| Body Mass Index >30 kg/m^2^ | 1.473 | 1.059 – 2.049 | 0.02 | 1.497 | 1.034 – 2.167 | 0.03 |
| EBV-Positive | 0.735 | 0.525 – 1.023 | 0.07 | 0.633 | 0.421 – 0.950 | 0.03 |
| PV Thrombosis | 2.347 | 1.528 – 3.606 | <0.001 | 2.448 | 1.497 – 4.004 | <0.001 |
| Ventilator Support | 2.879 | 1.940 – 4.272 | <0.001 | 3.562 | 2.304 – 5.506 | <0.001 |
| Etiology of ESLD |  |  | 0.46 |  |  | 0.45 |
| Acute | Reference |  |  | Reference |  |  |
| CC/NASH | 0.717 | 0.280 – 1.841 |  | 0.479 | 0.172 – 1.333 |  |
| Cholestatic | 0.862 | 0.323 – 2.296 |  | 0.771 | 0.267 – 2.227 |  |
| Cirrhosis (NOS) | 0.941 | 0.323 – 2.296 |  | 0.654 | 0.215 – 1.987 |  |
| Congenital/Metabolic | 0.670 | 0.207 – 2.172 |  | 0.585 | 0.162 – 2.111 |  |
| Alcohol | 0.693 | 0.277 – 1.737 |  | 0.642 | 0.239 – 1.724 |  |
| HBV | 1.313 | 0.398 – 4.234 |  | 1.499 | 0.423 – 5.314 |  |
| HCV | 0.502 | 0.204 – 1.237 |  | 0.478 | 0.181 – 1.262 |  |
| HCC | 0.838 | 0.317 – 2.16 |  | 0.637 | 0.220 – 1.849 |  |
| Other | 0.656 | 0.074 – 5.846 |  | n/a |  |  |
| Region of Transplant |  |  | 0.96 |  |  | 0.73 |
| 1 | Reference |  |  | Reference |  |  |
| 2 | 0.727 | 0.313 – 1.687 |  | 0.560 | 0.223 – 1.402 |  |
| 3 | 1.177 | 0.483 – 2.870 |  | 0.958 | 0.362 – 2.531 |  |
| 4 | 1.066 | 0.421 – 2.696 |  | 0.834 | 0.295 – 2.362 |  |
| 5 | 0.769 | 0.335- 1.766 |  | 0.505 | 0.202 – 1.267 |  |
| 6 | 0.951 | 0.299 – 3.023 |  | 0.991 | 0.292 – 3.367 |  |
| 7 | 0.833 | 0.357 – 1.947 |  | 0.697 | 0.276 – 1.764 |  |
| 8 | 0.969 | 0.394 – 2.384 |  | 0.879 | 0.327 – 2.365 |  |
| 9 | 1.033 | 0.420 – 2.542 |  | 0.823 | 0.309 – 2.189 |  |
| 10 | 1.137 | 0.411 – 3.147 |  | 1.067 | 0.361 – 3.147 |  |
| 11 | 0.835 | 0.331 – 2.106 |  | 0.635 | 0.225 – 2.655 |  |

Table S5B. Logistic Regression Modeling for 90-Day Graft Loss in Higher MELD Recipients

|  | Unadjusted | | | Adjusted^a^ | | |
| --- | --- | --- | --- | --- | --- | --- |
|  | OR | 95% CI | p-Value | OR | 95% CI | p-Value |
| Donor Variables |  |  |  |  |  |  |
| Age Groups |  |  | <0.001 |  |  | 0.003 |
| <35 Years | Reference |  |  | Reference |  |  |
| 35 to 44 Years | 1.794 | 1.056 – 3.046 |  | 1.467 | 0.824 – 2.614 |  |
| 45 to 54 Years | 1.712 | 1.050 – 2.793 |  | 1.572 | 0.931 – 2.654 |  |
| 55 to 64 Years | 2.707 | 1.684 – 4.351 |  | 2.509 | 1.503 – 4.190 |  |
| ≥65 Years | 2.399 | 1.395 – 4.124 |  | 2.231 | 1.229 – 4.050 |  |
| Body Mass Index >30 kg/m^2^ | 1.386 | 1.058 – 1.815 | 0.02 | 1.356 | 1.002 – 1.834 | 0.049 |
| Biopsy Result- % MaS |  |  | 0.007 |  |  | 0.001 |
| 0 to 9% | Reference |  |  | Reference |  |  |
| 10 to 19% | 1.112 | 0.770 – 1.607 |  | 1.017 | 0.684 – 1.514 |  |
| 20 to 29% | 1.272 | 0.746 – 2.171 |  | 1.337 | 0.763 – 2.344 |  |
| 30 to 39% | 1.817 | 1.002 – 3.296 |  | 1.846 | 0.919 – 3.708 |  |
| 40 to 49% | 4.904 | 2.340 – 10.279 |  | 6.687 | 3.024 – 14.790 |  |
| 50 to 59% | 1.709 | 0.379 – 7.716 |  | 1.659 | 0.339 – 8.099 |  |
| ≥60% | 0.684 | 0.090 – 5.215 |  | 0.613 | 0.076 – 8.099 |  |
| Prior MI | 1.616 | 0.962 – 2.714 | 0.08 | 1.851 | 1.039 – 3.295 | 0.046 |
|  |  |  |  |  |  |  |
| Cold Ischemic Time |  |  | <0.001 |  |  | <0.001 |
| <8 Hours | Reference |  |  | Reference |  |  |
| 8 to 12 Hours | 1.710 | 1.282 – 2.282 |  | 1.714 | 1.241 – 2.367 |  |
| ≥12 Hours | 2.690 | 1.465 – 4.941 |  | 3.471 | 1.750 – 6.882 |  |
| Recipient Variables |  |  |  |  |  |  |
| Age Groups |  |  | 0.02 |  |  | 0.07 |
| <35 Years | Reference |  |  | Reference |  |  |
| 35 to 44 Years | 0.770 | 0345 – 1.713 |  | 0.687 | 0.288 – 1.640 |  |
| 45 to 54 Years | 1.067 | 0.550 – 2.069 |  | 1.078 | 0.526 – 2.212 |  |
| 55 to 64 Years | 1.121 | 0.583 – 2.153 |  | 1.014 | 0.498 – 2.065 |  |
| ≥65 Years | 1.938 | 0.957 – 3.927 |  | 1.770 | 0.819 – 3.829 |  |
| EBV-Positive | 0.755 | 0.575 – 0.990 | 0.04 | 0.605 | 0.436 – 0.841 | 0.003 |
| PV Thrombosis | 2.455 | 1.718 – 3.510 | <0.001 | 2.614 | 1.743 – 3.919 | <0.001 |
| Ventilator Support | 2.384 | 1.689 – 3.363 | <0.001 | 2.734 | 1.872 – 3.992 | <0.001 |
| Etiology of ESLD |  |  | 0.69 |  |  | 0.93 |
| Acute | Reference |  |  | Reference |  |  |
| CC/NASH | 0.919 | 0.408 – 2.070 |  | 0.688 | 0.289 – 1.641 |  |
| Cholestatic | 0.843 | 0.355 – 1.999 |  | 0.710 | 0.282 – 1.792 |  |
| Cirrhosis (NOS) | 0.980 | 0.404 – 2.376 |  | 0.697 | 0.266 – 1.829 |  |
| Congenital/Metabolic | 0.847 | 0.316 – 2.268 |  | 0.772 | 0.267 – 2.231 |  |
| Alcohol | 0.734 | 0.329 – 1.639 |  | 0.661 | 0.281 – 1.557 |  |
| HBV | 1.326 | 0.463 – 3.797 |  | 1.315 | 0.427 – 4.052 |  |
| HCV | 0.675 | 0.309 – 1.473 |  | 0.616 | 0.268 – 1.419 |  |
| HCC | 1.014 | 0.438 – 2.347 |  | 0.707 | 0.285 – 1.756 |  |
| Other | 1.017 | 0.195 – 5.290 |  | 0.639 | 0.071 – 5.776 |  |
| Region of Transplant |  |  | 0.41 |  |  | 0.07 |
| 1 | Reference |  |  | Reference |  |  |
| 2 | 0.740 | 0.377 – 1.449 |  | 0.527 | 0.252 – 1.103 |  |
| 3 | 1.063 | 0.513 – 2.202 |  | 0.878 | 0.397 – 1.942 |  |
| 4 | 1.038 | 0.491 – 2.196 |  | 0.784 | 0.338 – 1.816 |  |
| 5 | 0.622 | 0.314 – 1.231 |  | 0.383 | 0.179 – 0.817 |  |
| 6 | 0.679 | 0.246 – 1.872 |  | 0.683 | 0.235 – 1.985 |  |
| 7 | 0.785 | 0.396 – 1.556 |  | 0.646 | 0.306 – 1.363 |  |
| 8 | 0.792 | 0.376 – 1.669 |  | 0.645 | 0.285 – 1.464 |  |
| 9 | 1.258 | 0.622 – 2.548 |  | 1.013 | 0.472 – 2.174 |  |
| 10 | 1.144 | 0.505 – 2.596 |  | 1.095 | 0.456 – 2.630 |  |
| 11 | 0.852 | 0.407 – 1.783 |  | 0.668 | 0.294 – 1.520 |  |

Table S5C. Logistic Regression Modeling for 1-Year Graft Loss in Higher MELD Recipients

|  | Unadjusted | | | Adjusted^a^ | | |
| --- | --- | --- | --- | --- | --- | --- |
|  | OR | 95% CI | p-Value | OR | 95% CI | p-Value |
| Donor Variables |  |  |  |  |  |  |
| Age Groups |  |  | <0.001 |  |  | <0.001 |
| <35 Years | Reference |  |  | Reference |  |  |
| 35 to 44 Years | 1.380 | 0.922 – 2.065 |  | 1.305 | 0.838 – 2.032 |  |
| 45 to 54 Years | 1.502 | 1.044 – 2.160 |  | 1.590 | 1.071 – 2.361 |  |
| 55 to 64 Years | 2.439 | 1.712 – 3.473 |  | 2.631 | 1.784 – 3.881 |  |
| ≥65 Years | 1.973 | 1.306 – 2.981 |  | 2.117 | 1.345 – 3.333 |  |
| Biopsy Result- % MaS |  |  | 0.07 |  |  | 0.01 |
| 0 to 9% | Reference |  |  | Reference |  |  |
| 10 to 19% | 1.064 | 0.798 – 1.418 |  | 1.015 | 0.745 – 1.383 |  |
| 20 to 29% | 1.364 | 0.904 – 2.058 |  | 1.426 | 0.923 – 2.201 |  |
| 30 to 39% | 1.142 | 0.670 – 1.946 |  | 1.151 | 0.656 – 2.019 |  |
| 40 to 49% | 2.924 | 1.401 – 6.101 |  | 3.986 | 1.841 – 8.634 |  |
| 50 to 59% | 2.058 | 0.629 – 6.734 |  | 2.720 | 0.756 – 9.785 |  |
| ≥60% | 0.356 | 0.046 – 2.735 |  | 0.355 | 0.045 – 2.827 |  |
|  |  |  |  |  |  |  |
| Cold Ischemic Time |  |  | 0.03 |  |  | 0.03 |
| <8 Hours | Reference |  |  | Reference |  |  |
| 8 to 12 Hours | 1.304 | 1.032 – 1.646 |  | 1.348 | 1.038 – 1.749 |  |
| ≥12 Hours | 1.698 | 0.978 – 2.949 |  | 1.816 | 0.973 – 3.393 |  |
| Recipient Variables |  |  |  |  |  |  |
| Age Groups |  |  | <0.001 |  |  | <0.001 |
| <35 Years | Reference |  |  | Reference |  |  |
| 35 to 44 Years | 0.694 | 0.362 – 1.334 |  | 0.742 | 0.359 – 1.533 |  |
| 45 to 54 Years | 1.127 | 0.659 – 1.927 |  | 1.261 | 0.686 – 2.318 |  |
| 55 to 64 Years | 1.208 | 0.712 – 2.051 |  | 1.169 | 0.639 – 2.135 |  |
| ≥65 Years | 2.398 | 1.353 – 4.250 |  | 2.379 | 1.245 – 4.548 |  |
| EBV-Positive | 0.816 | 0.658 – 1.013 | 0.07 | 0.694 | 0.535 – 0.900 | 0.006 |
| PV Thrombosis | 2.027 | 1.481 – 2.775 | <0.001 | 1.973 | 1.389 – 2.803 | 0.002 |
| Dialysis prior to Txp | 1.423 | 1.146 – 1.767 | 0.002 | 1.444 | 1.123 – 1.856 | 0.17 |
| Ventilator Support | 2.344 | 1.756 – 3.128 | <0.001 | 2.328 | 1.676 – 3.233 | <0.001 |
| Etiology of ESLD |  |  | 0.10 |  |  | 0.17 |
| Acute | Reference |  |  | Reference |  |  |
| CC/NASH | 1.354 | 0.655 – 2.801 |  | 1.029 | 0.478 – 2.218 |  |
| Cholestatic | 1.147 | 0.532 – 2.475 |  | 0.939 | 0.415 – 2.126 |  |
| Cirrhosis (NOS) | 1.513 | 0.691 – 3.312 |  | 1.301 | 0.565 – 2.999 |  |
| Congenital/Metabolic | 1.275 | 0.543 – 2.993 |  | 1.198 | 0.480 – 2.989 |  |
| Alcohol | 0.963 | 0.467 – 1.986 |  | 0.867 | 0.405 – 1.856 |  |
| HBV | 1.754 | 0.690 – 4.458 |  | 1.975 | 0.734 – 5.314 |  |
| HCV | 1.235 | 0.615 – 2.481 |  | 1.212 | 0.582 – 2.523 |  |
| HCC | 1.957 | 0.937 – 4.087 |  | 1.690 | 0.776 – 3.680 |  |
| Other | 1.754 | 0.475 – 6.479 |  | 1.380 | 0.308 – 6.173 |  |
| Region of Transplant |  |  | 0.72 |  |  | 0.36 |
| 1 | Reference |  |  | Reference |  |  |
| 2 | 0.778 | 0.461 – 1.310 |  | 0.657 | 0.365 – 1.181 |  |
| 3 | 1.028 | 0.578 – 1.828 |  | 0.933 | 0.493 – 1.768 |  |
| 4 | 0.822 | 0.446 – 1.515 |  | 0.647 | 0.323 – 1.296 |  |
| 5 | 0.636 | 0.375 – 1.080 |  | 0.484 | 0.265 – 0.883 |  |
| 6 | 0.645 | 0.278 – 1.497 |  | 0.714 | 0.291 – 1.752 |  |
| 7 | 0.819 | 0.481 – 1.392 |  | 0.747 | 0.413 – 1.354 |  |
| 8 | 0.806 | 0.452 – 1.439 |  | 0.789 | 0.412 – 1.512 |  |
| 9 | 0.960 | 0.544 – 1.696 |  | 0.866 | 0.462 – 1.624 |  |
| 10 | 0.796 | 0.403 – 1.576 |  | 0.766 | 0.365 – 1.610 |  |
| 11 | 0.789 | 0.441 – 1.412 |  | 0.740 | 0.384 – 1.426 |  |
